# Potential Applications of PRP-Enhanced Polybutylene Succinate Graft as Vascular Access for Chemotherapy in Oncological Patients: A Systematic Review

**DOI:** 10.3390/jfb16060228

**Published:** 2025-06-19

**Authors:** Andrea Gottardo, Giulia Bonventre, Tancredi Didier Bazan Russo, Pietro Zanatta, Giulia Lo Monte, Valerio Gristina, Antonio Galvano, Antonio Russo, Attilio Ignazio Lo Monte

**Affiliations:** 1 Precision Medicine in Medical, Surgical and Critical Care Area (Me.Pre.C.C.) Department, University of Palermo, 90127 Palermo, Italy; andrea.gottardo@unipa.it (A.G.); giulia.bonventre@unipa.it (G.B.); tancredididier.bazanrusso@unipa.it (T.D.B.R.); pzanatta25@gmail.com (P.Z.); valerio.gristina@unipa.it (V.G.); antonio.russo@usa.net (A.R.); attilioignazio.lomonte@unipa.it (A.I.L.M.); 2 Independent Researcher, 90036 Misilmeri, Italy; giulialom@virgilio.it

**Keywords:** polybutylene succinate, PBS, platelet-rich plasma, PRP, vascular graft, vascular access device, chemotherapy, oncology tissue engineering, regenerative medicine

## Abstract

This systematic review aimed to evaluate the potential of combining platelet-rich plasma (PRP) and polybutylene succinate (PBS) for the development of vascular grafts in patients undergoing chemotherapy. Relevant articles published in English or Italian were selected through a comprehensive search of MEDLINE (via PubMed) and the Cochrane Library. A total of ten screened articles and two additional relevant studies were included. The synthesis of results was conducted using digital tools, thoroughly reviewed by the authors. The quality assessment of the included studies revealed a medium-to-high risk of bias, with frequent limitations such as small sample sizes, experimental designs, and overall moderate to low methodological quality. Despite the heterogeneity of the findings, the available evidence suggests that radiocephalic graft placement and the use of PBS as a scaffold material, in combination with the growth factors contained in PRP, may improve clinical outcomes and reduce complications related to arteriovenous graft implantation. While promising, the current literature on this topic remains scarce and fragmented, underscoring the need for additional preclinical and clinical research. The proposed approach appears to hold potential for improving vascular access in oncology, but further in vivo validation is essential. This study received no external funding. Registration: PROSPERO ID CRD42025646724.

## 1. Background and Rationale

Safe access for chemotherapy (CT) in oncological clinical practice is essential. Unfortunately, many CT agents are toxic, leading to irritation, inflammation, and sclerosis of the venous walls. To mitigate peripheral vein damage, alternative administration devices are developed, namely central venous catheters (CVCs). These (among which the most known are the peripherally inserted central catheters (PICC) and the totally implanted port-a-cath) are inserted into larger, central veins, thus preserving peripheral ones.

Still, since CT solutions have high osmolarity, they draw water from vascular endothelium and the surrounding tissues. This further increases local damage, leading to infiltration or phlebitis, if CT is infused uncontrollably, even through CVCs. Therefore, CT administration must be regulated by an infusion pump, to precisely control doses and rates. This reduces the risk of overdoses, too-fast, or too-slow administrations that could lead to local damage and further side effects.

Nevertheless, the optimal administration device is still debated [1]. Indeed, their placement may lead to bloodstream infections [2], thrombosis [3], skin complications [4], migration [5], catheter fragment displacement [6], pneumothorax, nerve or arterial puncture [7], and paradoxical embolism following catheter removal [8].

**Figure 1:**
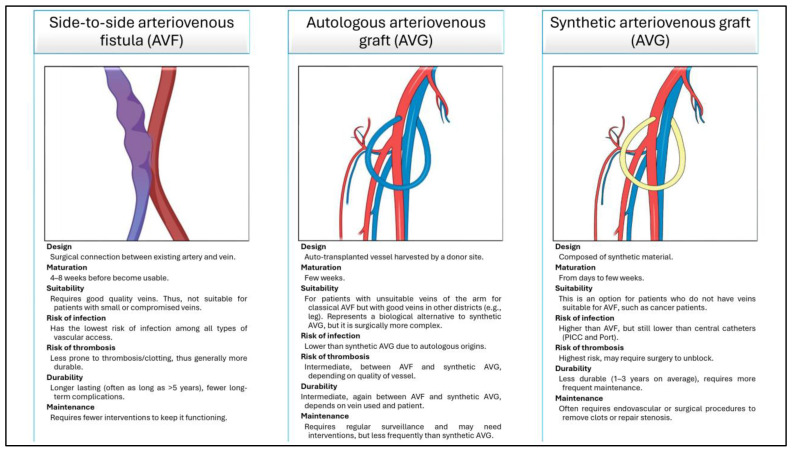
Graphical representation of all the possible layouts of peripheral venous accesses, with schematic details reported for each of them.

To reduce these complications, and when central venous access cannot be established, surgeons may opt for the creation of a peripherical venous access, the most common of which are the side-to-side arteriovenous fistula (AVF), the autologous arteriovenous graft (AVG), and the synthetic AVG. All of these are depicted and detailed in (Figure 1). Notably, these are usually created for patients requiring dialysis, not CT [9]. Nonetheless, since they allow high-flow vascular access to be obtained, several authors successfully adopted these approaches to facilitate CT passage during treatments [10,11].

Switching to the proper rationale of this research, an AVG in platelet-rich plasma (PRP)-enhanced polybutylene succinate (PBS) is hypothesized, to provide effective venous access for CT. This enhancement promises to fasten AVG endothelialization, while maintaining the original PBS characteristics. This forms the basis of the research hypothesis, to be evaluated with a systematic review of the literature.

PBS is a versatile synthetic polymer that is found applications in various fields of tissue engineering and regenerative medicine (TERM). Indeed, it can be fine-tuned to achieve desired mechanical and biological properties. Furthermore, being biocompatible and biodegradable, it can be reabsorbed without causing adverse reactions [12].

Platelets, otherwise, are blood components involved in both coagulation and tissue repair. These can be used in the form of platelet derivatives (PDs) (the most well-known of which is PRP), to promote healing processes. Indeed, platelets release numerous growth factors (GFs) that are crucial in tissue regeneration processes, guiding the growth and repair of damaged tissues [13,14,15]. In their praiseful work, Everts and colleagues [16] list a few platelet GFs, such as platelet-derived growth factor (PDGF), transforming growth factor (TGF), vascular endothelial growth factor (VEGF), epidermal growth factor (EGF), fibroblast growth factor (FGF), connective tissue growth factor (CTGF), insulin-like growth factor (IGF), hepatocyte growth factor (HGF), keratinocyte growth factor (KGF), angiopoietin-1 (Ang-1), platelet factor 4 (PF4), stromal cell derived factor (SDF-1α), and tumor necrosis factor (TNF).

Notably, such factors can be embedded onto the surfaces of a given scaffold by simply soaking it with PDs.

Since PRP-enhanced scaffolds are becoming a common trend in scientific literature [17,18], this paper seeks to evaluate if they may lead to a better endothelialization of vascular scaffolds. To do so, both in vivo and in vitro studies will be systematically assessed, aiming to find evidence to test this hypothesis.

## 2. Methods

### 2.1. PRISMA 2020 Checklist

This systematic review was conducted in accordance with the PRISMA 2020 Checklist, i.e., a 27-item statement aimed at improving the transparency, reproducibility, and completeness of reporting. The detailed checklist is available in (Supplementary Materials—Table S1). There, each item is mapped to the corresponding section in the manuscript, allowing readers to verify compliance with PRISMA guidelines (https://www.prisma-statement.org/ accessed on 28 February 2025). Items that were not applicable (e.g., meta-analysis procedures) are clearly marked. This approach ensures the review adheres to international reporting standards.

### 2.2. Preliminary Research Results, Rationale for the Systematic Review, and Research Diagram

In autumn 2024, preliminary research on the MEDLINE (via PubMed) and Cochrane Library was conducted, to find evidence regarding the joint use of PRP and PBS scaffolds. Even by broadening the search to any medical application, nothing could be traced in this regard. Therefore, a different approach was pursued, i.e., to carry out four mini research phases. These were always systematic, but separate and parallel, aimed at finding scientific evidence to be further gathered and analyzed. The four topics were as follows:

1. PDs for vascular reconstruction.
2. PBS for vascular reconstruction.
3. PBS + PDs.
4. Vascular graft or fistula for CT.

**Figure 2:**
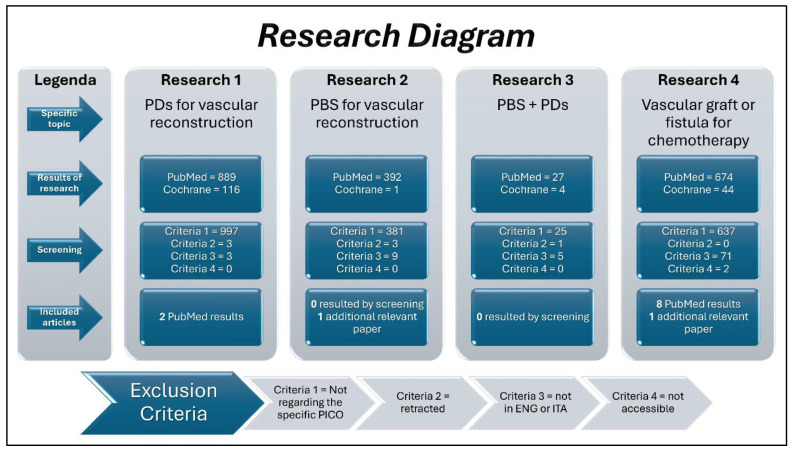
This Research Diagram reports the information commonly found in the canonical PRISMA 2020 Flow Diagrams, but by combining four of them (one for each research) into a single image. In fact, as reported in the “legend” box, for each research phase (characterized, precisely, by a “specific topic”), the number of results obtained from each source, the articles excluded during the screening phase according to the selected criteria, and the articles included are indicated. Finally, the diagram below shows the details of the four exclusion criteria.

The Research Diagram (Figure 2) helps to understand the proposed rationale. It resembles the PRISMA 2020 Flow Diagram, but is differently structured to better represent the actual research pattern.

### 2.3. Eligibility Criteria and Report of Research

In (document S1), all the research phases depicted in (Figure 2) are fully reported. This was performed by two authors (G.B. + A.Go.) and further evaluated by a third author (A.I.L.M.).

### 2.4. Data Extraction and Interpretation

Initially, a single author (A.Go.) summarized all the included articles using the Artificial Intelligence (AI) tool ChatGPT model 4o. Then, he manually reworked such drafts to highlight the rationale, results, and salient conclusions. The result was manually rechecked by two authors (G.B. + A.Ga.). Next, details about the technologies used are extracted, to analyze and compare them. Two authors (A.Go. + G.B.) carried out this endeavor, further evaluated by a third author (A.Ga.).

### 2.5. Criteria for Quality Assessment (QA)

Quality assessment was manually and independently conducted by two authors (A.Go. + G.B.) and further evaluated by a third author (A.Ga.). Particularly, since reviews along with both preclinical and clinical studies are included, only the Modified presentation of the Oxford Centre for Evidence-Based Medicine (OCEBM) levels of evidence scale [19] is adopted for any article. It assesses the grade of recommendation (with A representing the maximum grade and D the minimum) and level of evidence (with 1 representing the maximum level and 5 the minimum) by the type of study. Then, to further evaluate clinical studies, the Study Quality Assessment Tools made by National Institutes of Health—National Heart, Lung, and Blood Institute (NIH-NHLBI) [20] are used, particularly the Quality Assessment Tool of Case-Control Studies and the Quality Assessment Tool for Case Series Studies. In applying these two tools, 1 point was awarded for “yes” and 0 points for “no” or “other”; so, the former had a score range of 0 to 12, while the latter of 0 to 9.

## 3. Results

### 3.1. Table of Results and QA

Table 1 shows all the twelve included studies. Note that two additional relevant papers were identified through personal knowledge and expertise (as reported in the second column of the table), and that no studies are found regarding the joint use of PDs and PBS. Moreover, as reported in the last column, the average quality was low. Thankfully, this was due to the type of studies (indicated by the OCEBM scale) rather than to their quality (indicated by the NIH-NHLBI tools).

### 3.2. Synthesis of Results

In 1977, Bell et al. [21] (QA = C-4; 7/9) investigated the feasibility of creating subcutaneous AVFs in the upper limbs of five patients with hematological malignancies, all with impaired peripheral access due to extensive CT treatments. AVFs were attempted via direct arteriovenous (AV) anastomosis, while the AV grafting was attempted via mandril or Dacron grafts. All attempts failed due to early thrombosis or loss of pulse, likely secondary to chronic phlebitis and fibrotic venous strictures induced by the previous long-lasting chemotherapies. Histological examination in one patient confirmed extensive venous fibrosis and lumen obliteration. The authors concluded that late-stage AVF creation is largely ineffective in this population, recommending earlier intervention to avoid venous damage on the intended limb.

A year later, Levey et al. [22] (QA = C-4; 5/9) reported on 77 vascular access procedures performed in 74 pediatric patients (including infants < 10 kg) with hematological malignancies, solid tumors, and aplastic anemia. The primary indication was prolonged CT and transfusion support in patients with exhausted peripheral veins. The preferred technique was a saphenous vein loop fistula (n = 46), followed by femoral–femoral Thomas shunts (n = 16), bovine carotid heterografts (n = 5), and others. Most procedures were autologous; synthetic or biological grafts were used only when native veins were inadequate. Complications were infrequent: only four short-term and four long-term failures occurred (~10% combined), while nonfailure-causing complications occurred just six times. Among the latter, infections occurred just once, exclusively in a patient with a Thomas shunt. Saphenous fistulas showed high durability (up to 3 years), were well-tolerated even in immunocompromised hosts, and minimized the risks related to repeated venipuncture. The authors concluded that loop AVFs are a safe and effective solution for sustained vascular access in children undergoing intensive CT, and may significantly improve quality of care and comfort in pediatric oncology.

In 1979, Costantino et al. [23] (QA = C-4; 7/9) evaluated the use of upper-arm bovine AVGs in 48 cancer patients (51 grafts) requiring long-term CT. Particularly, 37 patients had myeloproliferative disorders and the remaining 11 had solid tumors; all presented with exhausted peripheral veins. The grafts were placed between the distal brachial artery and proximal brachial vein, often using just local anesthesia. The overall success rate was 81%, with a mean functional duration of 4.6 months (precisely, from 0 to 27 months). To note, the authors themselves underlined that this relatively brief span was more a function of patient fatality from cancer than failure of grafts. Thrombosis was the most common complication (31%), followed by infections (4%); reoperations were required in 35% of grafts, with a 44% salvage rate. No mortality was associated with the procedure, and both infection and hemorrhage rates remained low despite frequent leukopenia and some cases of thrombocytopenia. Patient and staff satisfaction was high. The authors concluded that upper-arm bovine AVGs offer a safe and durable option for vascular access in CT patients. Moreover, they hypothesized that in earlier patients, with lower AV exhaustion, a radiocephalic AVF might be tested too, to avoid the need for more expensive grafts.

In the same year, the first article by Raaf [24] (QA = C-4; 6/9) treated the implant of 42 PTFE or Dacron AVGs in 40 CT patients with inadequate peripheral veins. Most grafts (27/42) were placed in a straight configuration in the arms, with others in loop configurations in the arms (5/42) and legs (10/42). Except for one pediatric case, all procedures were conducted under local anesthesia, with follow-ups extending to 15 months. At study conclusion, 30 patients were still alive (31 grafts) and 10 were deceased (11 grafts): the former reported 26 functioning grafts, along with four grafts subject to thrombosis and one removed for suspected infection; conversely, the latter reported eight functioning grafts at the time of death, two grafts subjected to thrombosis and one removed for suspected infection. Unfortunately, the author only made assessments regarding the straight or loop configuration (with the former outperforming the latter). Moreover, the prosthesis material was chosen in advance for economic reasons (PTFE proved comparable but cheaper than Dacron). Nonetheless, the study concluded that AVGs, particularly the PTFE ones in straight configurations, are effective in providing vascular access in CT patients with compromised peripheral veins.

In 1981, Daly et al. [25] (QA = D-5) presented a comprehensive review of long-term vascular access options in oncology, contextualizing their own clinical experience with 63 PTFE or Dacron AVGs implanted in 60 cancer patients with sclerotic peripheral veins after prolonged CT. The article highlights the need for reliable and durable venous access in oncology, comparing externalized catheters, totally implanted ports, and AVGs in terms of infection, thrombosis, ease of use, and patient compliance. The authors stress that AVGs offer specific advantages—particularly for patients requiring frequent, long-term infusions—due to their subcutaneous placement and reduced maintenance. In their cohort, 58 PTFE grafts were placed in a straight (n = 43) or loop (n = 5) configuration in the upper limbs, or even in the thigh (n = 10, loop-configured). Early thrombosis occurred in 11 cases, late in eight, and infection in two (only one confirmed). Twenty-eight grafts retained patency without complications (mean duration = 6.3 months). The study concluded that AVGs can serve as effective and well-tolerated vascular access in cancer patients and should be considered as a viable alternative to CVCs.

In 1983, Wobbes and colleagues [10] (QA = C-4; 8/9) published the result of their five-year study, which rely on AVFs to reduce poly-CT-induced sclerosis of the veins. They primarily used end-to-side radiocephalic fistulas at the wrist, applied in 88 operations on 76 patients with a 64% success rate. Alternative elbow fistulas and LSV autografts were employed with unsuitable wrist sites, achieving functional success in 59% and 77% of cases, respectively. Both wrist and elbow AVF were implanted under local anesthesia, while all the other procedures were under general anesthesia. Despite all patients (except the leukemia ones with thrombocytopenia) receiving anticoagulants, early thrombosis resulted as the prevalent complication. AVF performances were influenced by preoperative CT duration: shorter treatment durations correlated with higher success rates for wrist fistulas; conversely, longer treatment correlated with higher success rates for elbow fistulas. Notably, attempts to use other techniques (e.g., PTFE grafts in the forearm) were less successful, suggesting a preference for natural AVFs over synthetic alternatives. The study concluded that, as for dialysis, AVFs can provide effective vascular access for CT, though thrombosis risks are higher in cancer patients due to altered coagulation profiles. Indeed, the reported deaths are primarily due to cancer and thrombosis. To overcome this issue, recommendations include using a continuous drip during surgery to prevent late thrombosis, and selecting the fistula location based on CT length to minimize early thrombosis.

Raaf’s second study [26] (QA = C-4; 6/9), published in 1985, reported the results obtained with 826 vascular access devices in 681 cancer patients over 7 years. Among them, 103 were PTFE AVGs, mainly placed in the upper limb for outpatient CT. Although there was no mortality and some grafts continued to function for more than 4 years, complications were relatively frequent: indeed, 43% of them reported complications, and 17% were lost for this reason. Particularly, 28% reported a thrombotic occlusion, and only half of them were successfully revised. Conversely, 698 silastic right atrial catheters (both single- and dual-lumen) demonstrated a significantly lower complication rate (19%) and no mortality. The 2.2-mm dual-lumen, right atrial catheter became the preferred device due to ease of insertion, versatility (including parenteral nutrition and blood draws), and lower thrombosis and infection rates—even in immunosuppressed individuals. Additional central devices included 13 HemoCath catheters for hemodialysis, and 12 Ports for outpatient CT. The author concluded that silastic right atrial catheters offer a safer, more manageable solution, especially in leukopenic or thrombocytopenic patients, though the choice of the proper device should be assessed case by case.

Moving closer to the present day, in 2007, Tewari et al. [11] (QA = B-3b; 3/12) tested again AVFs. Their study, however, differs from those seen previously: here, in fact, AVFs were crafted to improve the venous capacity of the hand and forearm, thus allowing CT administration into the dilated peripheral veins, not into the fistula itself. Ten CT-naïve patients underwent the AVFs creation under local anesthesia. Follow-up assessments included cardiac scores (linked to rare AVF complications), venous accessibility, number of needle pricks required during CT cycles, erythema, edema, thrombophlebitis, pigmentation, and skin necrosis. Over one year, this method proved successful: enhanced accessibility and reduction in prick numbers are reported, the failure rate drops to 20% and, especially, no complication is reported. Furthermore, the procedure required minimal hospital resources. The study concluded that wrist AVFs can significantly improve peripheral venous access for CT, reducing morbidity and costs while improving patient comfort.

The 2009 review by Cifarelli [27] (QA = C-5) compared tunneled CVCs and prosthetic AVGs for long-term vascular access, primarily in hemodialysis but with implications for oncology and frail patients. Over three decades, various and different synthetic, biological, and biosynthetic grafts have been developed to overcome native vein unavailability. Despite known drawbacks (such as venous anastomosis intimal hyperplasia) grafts are favored over CVCs, which are linked to higher infection risk, early central venous stenosis, and mortality. Indeed, large-scale studies (e.g., DOPPS [30]) show reduced mortality with AVGs compared to CVCs. However, CVCs remain indicated in select populations: pediatric patients <20 kg, individuals with severe cardiac dysfunction, terminal cancer patients, or those with peripheral vascular disease where grafting poses an ischemic risk. The author advocates for individualized access planning and prioritizing grafts over CVCs when clinically feasible, to preserve future options and reduce complications.

In 2014, Bertanha et al. [28] (QA = D-5) explored the construction of advanced blood vessel substitutes. Particularly, they wanted to recreate a homologous endothelium on decellularized heterologous vein scaffolds, using patient-derived MSCs induced by its own platelet GFs. To do so, adipose tissue and vena cava were harvested by fifteen adult rabbits, for MSC isolation and scaffold preparation, respectively. The vena cava was decellularized, seeded with MSCs and, in a test group, incubated with endothelial inductor growth factors (EIGFs)—namely, the supernatant obtained during the lysis of human platelets; that is, the soluble part of the PL itself. The in vitro results proved successful scaffold decellularization and efficient endothelial cell differentiation. Particularly, the latter resulted in significant improvement in the test group, underlying the platelet’s GF capabilities. Even histological analysis showed proper morphologic characteristics and protein expression pattern, further suggesting effective endothelial functionality. The study concluded that such a scaffold provides a promising method for developing biocompatible vascular grafts. Future perspectives were further seeding with smooth muscle cells and in vivo tests.

Miceli et al. [12] (QA = D-5) aimed to overcome the limitation of common-used biomaterials in producing small conduits for TERM. Therefore, in 2022, they tested PBS to craft electrospun, small-diameter tubular scaffolds, with an ECM-mimicking microfibrous structure. Particularly, they wanted the scaffold to support cell adhesion and growth, while preventing cell infiltration through the graft wall. The obtained scaffold, with just 2.62 mm of inner diameter and a homogeneous micro-porous structure, underwent thorough morphological, mechanical, and cytocompatibility assessments. Biomechanical testing demonstrated resistance to sutures, physiological fluid degradation, and various mechanical stressors. Moreover, in vitro tests show promising biointegrative capacity. Such results underscored the potential of PBS in developing AVGs, highlighting its ability to support cell growth while maintaining necessary mechanical characteristics. Furthermore, the authors suggest that PBS may be well-suited to fine-tuning, mass-production, shipping, and storing in both a reproducible and controlled way, and at a favorable cost.

Finally, in 2023, Li et al. [29] (QA = C-5) developed a small-diameter (<6 mm) tissue-engineered vascular graft (TEVG), which was further functionalized with PRP to improve its endothelialization and vascularization. In detail, electrospun TEVGs composed of poly-L-lactic acid (PLLA) and gelatin were infused with 2%, 5%, and 10% volume/volume (*v*/*v*) ratio of PRP-gel supernatant, obtaining the so-called PRP-TEVGs. These were tested both in vivo and in vitro using New Zealand rabbits and their carotid artery models, respectively. Preliminary in vitro tests showed significant cell growth stimulation with 2% and 5% PRP; conversely, 10% PRP reduced cell growth. Therefore, the following studies were carried out using the 5% V-V ratio. The following in vitro tests showed that such graft promoted proliferation, migration, and functional maturation of vascular endothelial cells (VECs) and smooth muscle cells (VSMCs). Moreover, probably thanks to the specific experimental process, platelet GFs (VEGF, PDGF, TGF-β1) were released continuously over 25 days, without a burst effect. Four-week in vivo tests proved 5% PRP-TEVGs superiority against nude TEVGs: indeed, statistically significant improvements were obtained regarding neointimal endothelial coverage, thickening of VSMC layers, increased expression of functional markers (e.g., eNOS), and higher neovascularization of the outer wall of the vessel. The study concluded that PRP-functionalized TEVGs significantly support vascular integration and remodeling, offering a promising strategy for improving small-caliber graft performance. Limitations included short-term follow-up and insufficient mechanical robustness, suggesting future studies in large animal models.

### 3.3. Summary of Results

The above clinical studies [10,11,21,22,23,24,26] indicate that, in patients with compromised peripheral veins, both AVFs and AVGs can serve as alternative vascular access for CT infusion. These approaches, generally performed under local or regional anesthesia, have demonstrated effectiveness in maintaining adequate blood flow and ensuring drug delivery. However, they are frequently associated with thrombosis, occlusion, or infection. Notably, most of the included studies are dated, with several published in the 1970s and 1980s, reflecting historical practices and device technologies that may not fully align with current standards. Nonetheless, the outcomes appear more favorable when AVGs are implanted earlier in the treatment course and when autologous materials are available. Loop configurations (e.g., saphenous AVF loops) were especially effective in pediatric and frail populations. Nonetheless, the risk of failure increases significantly when venous structures are already compromised. These studies support the theoretical validity of using such expedients for CT delivery, but also highlight the need to optimize graft configuration, timing of implantation, and material properties to improve medium- to long-term outcomes.

Regarding preclinical studies, the three included articles [12,28,29] tested heterogeneous strategies for vascular graft development. One study employed electrospun “nude” PBS scaffolds [12], while another developed more complex biological scaffolds by seeding MSCs onto decellularized rabbit veins, further enhanced with EIGFs [28]. A more recent work introduced a PRP-functionalized PLLA/gelatin AVG, which showed enhanced endothelialization and vascular regeneration in vitro and in vivo, although mechanical proprieties were suboptimal [29]. Therefore, while each approach demonstrated promising results, none directly tested PRP-enhanced PBS grafts. Thus, the current preclinical literature provides only support for both the positive structural proprieties of PBS as a scaffold material and the potential added value of PDs functionalization but lacks a unified model integrating both features.

Finally, the two reviews [25,27] offer broader insights into the clinical use of vascular access devices. Both highlight the drawbacks of CVCs, especially their higher risk of infection, central venous stenosis, and limited long-term patency, thus supporting the preferential use of AVGs when clinically appropriate. Cifarelli emphasized the importance of individualized access planning and endorsed AVGs as a more durable and safer option across chronic settings. Daly provided a comprehensive overview of vascular access strategies in oncology, underscoring the advantages of subcutaneous AVGs in terms of ease of maintenance and patient compliance, while reporting favorable outcomes from their own clinical cohort. Taken together, the reviews reinforce the rationale for developing optimized graft-based solutions that balance mechanical reliability, infection control, and usability in patients requiring prolonged infusion therapies.

### 3.4. Details About Adopted Technologies

In (Supplementary Materials—Table S2), all information regarding technical details and the overall results of each adopted technology (i.e., each device assessed in all the obtained papers) are listed. In the following lines, the results of the confront of that information are reported.

The studies analyzed in this section are mostly dated, reflecting the practices of past decades, and often present heterogeneous results. Comparisons between devices were limited and anecdotal, lacking the rigor of properly designed case–control studies. Nevertheless, the data extracted allow for a descriptive synthesis of the adopted technologies, highlighting key technical elements and clinical outcomes.

A wide range of vascular access modalities was reported. Briefly, the adopted technology found in the obtained papers were the following: generic CVCs; AVFs (side-to-side, end-to-side, loop, Thomas shunts); AVGs (autograft, allograft, bovine); synthetic AVGs (PTFE, Dacron); bioengineered AVGs (nude, functionalized).

CVCs were placed percutaneously or via the cutdown technique, mainly through the subclavian vein, and although high performance in repeated usage were reported, they require strict maintenance protocols and were associated with high infection and occlusion rates. Their use was suggested to fragile or pediatric patients, or when no other options were available [25,27].

AVFs were created through various anastomotic configurations: side-to-side AVFs showed promising outcomes with minimal complications in CT-naïve patients [11], while end-to-side AVFs demonstrated variable results depending on anatomical site and patient condition—ranging from 64% success in wrist AVFs to 0% in cases with severe CT-induced venous damage [10,21]. Loop AVFs, mostly reported in pediatric patients, showed high usability and low complication rates, especially when performed in the lower limbs [22]. Finally, Thomas shunts provided a viable alternative when upper limb access was unfeasible, with 13 out of 16 shunts functioning adequately [22].

AVGs were constructed using autologous, allogeneic, bovine, or synthetic materials. Autologous AVGs using LSV achieved a 77% success rate in forearm placement [10]. Allografts were rarely used and only in patients with no other access options [22]. Bovine-derived grafts yielded intermediate outcomes. While Levey reported no complications in a small sample [22], Costantino found thrombosis in 12 patients and infections in four, yet 81% of grafts remained functional at endpoint [23]; good histologic biocompatibility and aneurysm risk were noted in Cifarelli’s review [27]. Synthetic AVGs, especially those made of PTFE, were widely adopted and demonstrated acceptable long-term patency with complication rates up to 43%, including thrombosis and rare infections [24,25,26,27]. Dacron grafts were infrequently used and consistently not recommended compared to PTFE [21,24,25].

Bioengineered graft, assessed only in preclinical settings, included electrospun PBS-based nude scaffolds with good mechanical and cytocompatibility profiles [12], as well as functionalized AVGs incorporating platelet-derived growth factors or MSCs to enhance endothelialization and integration [28,29].

When comparing the technical complexity of the methods, CVCs and synthetic PTFE AVGs emerge as the most straightforward in terms of surgical execution and perioperative management. AVFs, particularly loop or end-to-side variants, require more surgical skill and vessel availability, while autologous or allogeneic AVGs involve additional procedures such as graft harvesting or preservation. Thomas shunts, although technically simple, are reserved for extreme cases. Among preclinical technologies, nude scaffolds are easier to manufacture and standardize, while functionalized models introduce additional steps in scaffold preparation and cell handling.

Regarding clinical effectiveness, autologous AVGs demonstrated the best reported outcomes (77% success), followed by loop AVFs (high usability, minimal complications) and wrist AVFs (64%). PTFE AVGs showed acceptable durability despite notable complication rates. CVCs had the worst (claimed) outcome profile due to infection and thrombosis. Among preclinical studies, functionalized scaffolds exhibited the most promising biological performance, including endothelial differentiation and vascular integration, though long-term data and translational validation remain lacking.

Together, these studies offer promising but heterogeneous models, although with no study directly testing PRP-enhanced PBS AVGs. To overcome current limitations, future research should focus on prospective comparative studies directly evaluating traditional techniques (AVFs, AVGs, CVCs) against emerging bioengineered grafts in well-defined patient populations. Functionalization strategies, particularly involving GFs or stem cells, warrant further validation in large-animal models with physiological resemblance to humans. Moreover, cost-effectiveness analyses and longitudinal studies on device performance, complication rates, turn-around-times (TAT), and quality of life (QoL) are essential to inform clinical decision-making and policy.

## 4. Discussion

Tissue engineering, regenerative medicine, and the use of biomaterials hold significant promises in modern medicine. One emerging yet underexplored strategy leverages functionalized vascular grafts for CT delivery, potentially reducing complications of both synthetic AVGs and autologous AVFs. Particularly, AVGs, while established, are plagued by limited long-term viability due to non-supportive endothelialization and vascularization, thus leading to thrombosis, infections, and other failures [31,32]. Similarly, AVFs, while reducing rejection and infection risks, are constrained by vessel availability and harvesting complications [33].

To overcome these issues, an alternative approach is hypothesized, i.e., a PRP-enhanced PBS graft, to serve as a bioactive vascular conduit. Such graft would be produced in two simple steps: electrospinning PBS to create the scaffold, to be soaked in PRP. This will combine PBS structural properties with PRP regenerative potential, enhancing endothelialization and biocompatibility while reducing complications. Therefore, the patient will benefit from a functionalized AVG that, once implanted, can quickly become a full-fledged blood vessel, specifically made for CT infusion—of course, always delivered with an infusion pump, to ensure optimal drug management, and under anticoagulant therapy, as per current clinical practice.

However, although promising, preliminary research revealed no clinical or preclinical studies in this regard. This prompted four mini systematic reviews, uncovering just twelve relevant studies, thus highlighting both the paucity of studies on the field and, conversely, the innovativeness of such a hypothesized solution to this unmet clinical need. Moreover, heterogeneity among studies made direct comparisons challenging, because each of them represented different “parts of the puzzle” in assembling.

Still, the gathered clinical evidence [10,11,21,22,23,24,25,26,27] offers a broader perspective. CVCs, though technically simple and rapidly deployable, seems to be associated with the highest rates of infection, thrombosis, and central venous stenosis, making them suitable only for restricted scenarios [25,27]. Conversely, autologous loop AVFs in pediatric patients [22] and radiocephalic AVFs [10] emerged as well-tolerated and effective options, particularly when used early in the treatment pathway. AVGs—both synthetic (e.g., PTFE) and biological (e.g., bovine or autologous vein)—showed acceptable patency rates but a high rate of thrombosis and reintervention, especially when used in advanced oncologic cases with poor vascular conditions [23,24,25,26]. Among the synthetic options, straight PTFE grafts outperformed loop configurations and Dacron alternatives [24,25]. Notably, only one study evaluated AVF creation not for direct infusion, but to enhance peripheral venous caliber and reduce complications from repeated needle punctures [11], providing interesting insight into alternative applications.

In contrast, preclinical studies [12,28,29] introduced more innovative strategies. Electrospun PBS scaffolds proved to be structurally robust, cytocompatibile, and easily producible [12], while functionalization with PDs or stem cells enhanced endothelialization and vascular integration in animal models [28,29]. The use of PRP appeared particularly promising in promoting neointima formation, cell proliferation, and neovascularization, although mechanical performance and follow-up duration remain as limitations.

Notably, two distinct bioengineering approaches were identified: on one side, nude biopolymeric scaffolds, such as those based on PBS, offer mechanical stability, tunable degradation, and cost-effective scalability. Their acellular composition simplifies production and regulatory handling, making them suitable for rapid manufacturing and storage. However, their lack of intrinsic biological cues may limit early host integration, relying on post-implantation colonization to achieve functional remodeling.

On the other side, biologically functionalized scaffolds—either seeded with MSCs or enriched with platelet-derived growth factors—are designed to actively modulate the microenvironment, promoting faster endothelialization, reduced thrombogenicity, and improved biointegration. Furthermore, if autologous material is used, potential immunologic responses might be limited. Therefore, these devices may better mimic native tissue behavior, yet they involve more complex, time-intensive preparation protocols and potential variability in cell-based components. As such, while offering enhanced regenerative potential, their clinical scalability and reproducibility remain challenging.

Nonetheless, both strategies showed favorable in vitro or short-term in vivo outcomes, underscoring the need for head-to-head comparisons in preclinical models. Particularly, future analyses should consider not only biological performance but also production timelines, costs, and regulatory implications to guide clinical translation.

In this context, PRP-enhanced PBS grafts have been hypothesized as a hybrid strategy that merges the mechanical advantages of synthetic polymeric scaffolds with the biological activity of autologous PDs. The rationale for this approach lies in the potential of PRP to promote inflammation, angiogenesis and cellular recruitment, facilitating rapid graft biointegration through a functionalization performed immediately prior to implantation. Indeed, unlike cellularized scaffolds, which require labor-intensive and time-consuming in vitro incubation, PRP-based functionalization can be achieved intraoperatively, as PRP preparation from autologous blood is feasible within 30 min [34,35] and requires only a brief soaking step. This strategy could, in theory, preserve the structural properties of PBS scaffolds—such as mechanical resistance and flexibility [12]—while enhancing local tissue responses through bioactive stimulation.

However, it must be emphasized that this combination has not yet been described or directly evaluated in the literature included in the present systematic review. The nearest available studies have examined PBS scaffolds in isolation [12] or explored the application of PDs in conjunction with biologically derived materials [28,29], but no study to date has investigated the integration of PRP with PBS grafts specifically. Therefore, the proposed approach remains theoretical and unvalidated, although the consistency of positive outcomes across related yet distinct experimental models supports the plausibility of its future development.

Several limitations must be acknowledged when interpreting the findings of this endeavor. First, the literature on this topic is limited and highly heterogeneous. Second, the included studies span a broad historical range, with many published in the 1970s and 1980s, reflecting clinical practices and materials no longer widely adopted. Third, the systematic review process was constrained by the lack of comparative studies and the absence of randomized trials. Finally, the overall methodological quality of the studies was strictly moderate, limiting the strength of the conclusions.

Nonetheless, this work consolidates a dispersed body of literature into a coherent framework and offers what appears to be the first structured assessment of PRP-enhanced PBS grafts as a theoretical solution for CT-related vascular access. It also revives a long-standing clinical challenge—first raised more than half a century ago, in 1971, by Bell and colleagues [21]—that remains unresolved to this day.

Future investigations, beginning with animal studies and progressing to controlled clinical trials, are strongly warranted to further explore this innovative application of TERM in oncology. In doing so, the entry of such devices into current clinical practice can be accelerated, should the results confirm expectations.

## 5. Conclusions and Future Direction

PRP-enhanced PBS AVGs represent a compelling but still hypothetical solution for improving vascular access in oncological patients requiring long-term CT. While both components—PBS scaffolds and PD bioactivation—have individually shown promise in preclinical settings, no current study has investigated their combination directly. Therefore, clinical applicability remains entirely speculative, and this paper should be intended as a state-of-the-art analysis, rather than a collection of evidence in favor of the implementation of such scaffolds within current clinical practice.

In summary, the findings of this systematic review highlight the fragmentation of existing evidence and the predominance of dated, low-to-moderate quality studies. Nevertheless, they provide a conceptual foundation upon which future research can build. In particular, PRP functionalization appears to be a practical, cost-effective, and intraoperatively feasible strategy to enhance synthetic graft integration, offering advantages over more complex cellularization protocols.

Future research should include

- Comparative in vivo studies assessing PRP-functionalized versus unmodified PBS scaffolds.
- Long-term follow-up in large-animal models, ideally with vascular anatomy closer to humans.
- Head-to-head comparisons between PRP-enhanced scaffolds and conventional AVGs or AVFs.
- Economic and cost–benefit analyses to inform translational viability.

Until these studies are completed, clinical implementation remains premature. Nonetheless, if future results confirm current hypotheses, PRP-enhanced PBS grafts may become a valuable option for durable, functional, and biocompatible vascular access in both oncology and other chronic infusion settings.

## Figures and Tables

**Table 1:** Table of Results.

First Author (Year) [Ref.]	Research (Source)	Topic	Type of Study	QA
S. N. Bell (1977) [21]	4—Vascular graft or fistula for CT (PubMed)	Subcutaneous AVFs and AVGs in patients with malignant hematologic disease presenting peripheral venous access complications for CT, antibiotic, and blood administration.	Human case series	Grade C, level 4; 7 points out of 9.
R. H. Levey (1978) [22]	4—Vascular graft or fistula for CT (PubMed)	Various AVFs and AVGs to facilitate and reduce complications of CT access for infants and children.	Human case series	Grade C, level 4; 5 points out of 9.
M. J. Costantino (1979) [23]	4—Vascular graft or fistula for CT (PubMed)	Creation of upper-arm bovine AVFs for CT.	Human case series	Grade C, level 4; 7 points out of 9.
J. H. Raaf (1979) [24]	4—Vascular graft or fistula for CT (PubMed)	Polytetrafluoroethylene (PTFE) AVGs in patients that require CT but suffer from damaged peripheral veins.	Human case series	Grade C, level 4; 6 points out of 9.
J. M. Daly (1981) [25]	4—Vascular graft or fistula for CT (PubMed)	AVFs, AVGs, and CVCs for vascular access in cancer patients.	Review	Grade D, level 5.
T. Wobbes (1983) [10]	4—Vascular graft or fistula for CT (PubMed)	Radiocephalic AVF, elbow AVF, and long saphenous vein (LSV) autografts in the inguinal region to give vascular access for CT in oncological patients.	Human case series	Grade C, level 4; 8 points out of 9.
J. H. Raaf (1985) [26]	4—Vascular graft or fistula for CT (PubMed)	PTFE AVGs versus classical vascular access devices for CT, antibiotic, and blood administration.	Human case series	Grade C, level 4; 6 points out of 9.
M. Tewari (2007) [11]	4—Vascular graft or fistula for CT (additional relevant paper)	Radiocephalic AVF in patients that require CT but with unattainable central venous access, compared with historical controls.	Human case-control study	Grade B, level 3b; 3 points out of 12.
M. Cifarelli (2009) [27]	4—Vascular graft or fistula for CT (PubMed)	Various AVGs versus CVCs for vascular access in cancer and dialysis patients.	Review	Grade D, level 5.
M. Bertanha (2014) [28]	1—PDs for vascular reconstruction (PubMed)	Rabbit-derived decellularized vein scaffold seeded with mesenchymal stem cells (MSCs) and incubated with the soluble part of the platelet lysate (PL) to stimulate the growth of new endothelium.	In vitro animal study	Grade D, level 5.
G. C. Miceli (2022) [12]	2—PBS for vascular reconstruction (additional relevant paper)	Biomechanical and in vitro tests of extracellular matrix (ECM)-mimicking PBS tubular scaffold for TERM.	In vitro human study	Grade D, level 5.
G. Li (2023) [29]	1—PDs for vascular reconstruction (PubMed)	In vivo and in vitro analysis of small diameter PRP-loaded tissue-engineered vascular grafts.	In vivo and in vitro animal study	Grade D, level 5.

## Data Availability

The original contributions presented in this study are included in the Supplementary Material. Further inquiries can be directed to the corresponding author.

## Supplementary Materials

The following supporting information can be downloaded at https://www.mdpi.com/article/10.3390/jfb16060228/s1, Table S1—PRISMA 2020 Checklist. Table S2—Details about Adopted Technology. Document S1—Search Strings, Eligibility Criteria, and Screening Phase.
