# Supplementary material for: Potential Applications of PRP-Enhanced Polybutylene Succinate Graft as Vascular Access for Chemotherapy in Oncological Patients: A Systematic Review
Source: J Funct Biomater. 2025 Jun 19;16(6):228. doi: 10.3390/jfb16060228 (PMC12193876; doi:10.3390/jfb16060228)
Supplement: Supplementary file 1 [file jfb-16-00228-s001.zip › jfb-3615150-supplementary.pdf]

Potential Applications of PRP-Enhanced Polybutylene Succinate Graft as Vascular Access for Chemotherapy in Oncological Patients: A Systematic Review

SUPPLEMENTARY MATERIALS

Table S1 – ***PRISMA 2020 Checklist***

Section and Topic	Item #	Checklist item	Location where item is reported
**TITLE**			
Title	1	Identify the report as a systematic review.	Title
**ABSTRACT**			
Abstract	2	See the PRISMA 2020 for Abstracts checklist.	Abstract & Graph. Ab.
**INTRODUCTION**			
Rationale	3	Describe the rationale for the review in the context of existing knowledge.	Sec. 1
Objectives	4	Provide an explicit statement of the objective(s) or question(s) the review addresses.	Sec. 1
**METHODS**			
Eligibility criteria	5	Specify the inclusion and exclusion criteria for the review and how studies were grouped for the syntheses.	Sec. 2.2+2.3 & Fig. 2
Information sources	6	Specify all databases, registers, websites, organisations, reference lists and other sources searched or consulted to identify studies. Specify the date when each source was last searched or consulted.	Sec. 2.2 & Fig. 2 & Supp.Mat.
Search strategy	7	Present the full search strategies for all databases, registers and websites, including any filters and limits used.	Fig. 2 & Supp.Mat.
Selection process	8	Specify the methods used to decide whether a study met the inclusion criteria of the review, including how many reviewers screened each record and each report retrieved, whether they worked independently, and if applicable, details of automation tools used in the process.	Sec. 2.3 & Supp.Mat.
Data collection process	9	Specify the methods used to collect data from reports, including how many reviewers collected data from each report, whether they worked independently, any processes for obtaining or confirming data from study investigators, and if applicable, details of automation tools used in the process.	Sec. 2.4 & Declarat.
Data items	10a	List and define all outcomes for which data were sought. Specify whether all results that were compatible with each outcome domain in each study were sought (e.g. for all measures, time points, analyses), and if not, the methods used to decide which results to collect.	Sec. 2.4 & Fig. 2 & Supp.Mat.
	10b	List and define all other variables for which data were sought (e.g. participant and intervention characteristics, funding sources). Describe any assumptions made about any missing or unclear information.	X
Study risk of bias assessment	11	Specify the methods used to assess risk of bias in the included studies, including details of the tool(s) used, how many reviewers assessed each study and whether they worked independently, and if applicable, details of automation tools used in the process.	Sec. 2.5
Effect measures	12	Specify for each outcome the effect measure(s) (e.g. risk ratio, mean difference) used in the synthesis or presentation of results.	X

Synthesis methods	13a	Describe the processes used to decide which studies were eligible for each synthesis (e.g. tabulating the study intervention characteristics and comparing against the planned groups for each synthesis (item #5)).	Sec.2.4 + All 3 & Tab.1+2
	13b	Describe any methods required to prepare the data for presentation or synthesis, such as handling of missing summary statistics, or data conversions.	X
	13c	Describe any methods used to tabulate or visually display results of individual studies and syntheses.	All Sec. 3 & Tab. 1+2
	13d	Describe any methods used to synthesize results and provide a rationale for the choice(s). If meta-analysis was performed, describe the model(s), method(s) to identify the presence and extent of statistical heterogeneity, and software package(s) used.	Sec. 2.4+3.1+3.3 & Tab.1+2
	13e	Describe any methods used to explore possible causes of heterogeneity among study results (e.g. subgroup analysis, meta-regression).	All Sec. 3
	13f	Describe any sensitivity analyses conducted to assess robustness of the synthesized results.	X
Reporting bias assessment	14	Describe any methods used to assess risk of bias due to missing results in a synthesis (arising from reporting biases).	X
Certainty assessment	15	Describe any methods used to assess certainty (or confidence) in the body of evidence for an outcome.	X
**RESULTS**			
Study selection	16a	Describe the results of the search and selection process, from the number of records identified in the search to the number of studies included in the review, ideally using a flow diagram.	Fig. 2 & Sec. 3.1 & Tab. 1
	16b	Cite studies that might appear to meet the inclusion criteria, but which were excluded, and explain why they were excluded.	X
Study characteristics	17	Cite each included study and present its characteristics.	Sec. 3.1 & Tab. 1
Risk of bias in studies	18	Present assessments of risk of bias for each included study.	Sec. 3.1+3.2 & Tab. 1
Results of individual studies	19	For all outcomes, present, for each study: (a) summary statistics for each group (where appropriate) and (b) an effect estimate and its precision (e.g. confidence/credible interval), ideally using structured tables or plots.	X
Results of syntheses	20a	For each synthesis, briefly summarise the characteristics and risk of bias among contributing studies.	Sec. 3.2
	20b	Present results of all statistical syntheses conducted. If meta-analysis was done, present for each the summary estimate and its precision (e.g. confidence/credible interval) and measures of statistical heterogeneity. If comparing groups, describe the direction of the effect.	X
	20c	Present results of all investigations of possible causes of heterogeneity among study results.	Sec. 3.4 + 4
	20d	Present results of all sensitivity analyses conducted to assess the robustness of the synthesized results.	X
Reporting biases	21	Present assessments of risk of bias due to missing results (arising from reporting biases) for each synthesis assessed.	X
Certainty of evidence	22	Present assessments of certainty (or confidence) in the body of evidence for each outcome assessed.	X
**DISCUSSION**			
Discussion	23a	Provide a general interpretation of the results in the context of other evidence.	Sec. 4
	23b	Discuss any limitations of the evidence included in the review.	Sec. 4
	23c	Discuss any limitations of the review processes used.	Sec. 4
	23d	Discuss implications of the results for practice, policy, and future research.	Sec. 4 + 5

OTHER INFORMATION			
Registration and protocol	24a	Provide registration information for the review, including register name and registration number, or state that the review was not registered.	Declarat.
	24b	Indicate where the review protocol can be accessed, or state that a protocol was not prepared.	Declarat.
	24c	Describe and explain any amendments to information provided at registration or in the protocol.	X
Support	25	Describe sources of financial or non-financial support for the review, and the role of the funders or sponsors in the review.	Declarat.
Competing interests	26	Declare any competing interests of review authors.	Declarat.
Availability of data, code and other materials	27	Report which of the following are publicly available and where they can be found: template data collection forms; data extracted from included studies; data used for all analyses; analytic code; any other materials used in the review.	Sec. 2.3 + Declarat. + Supp.Mat.

Table S2 – ***Details about Adopted Technologies***

Adopted technology	SUBTYPES [Ref.]<br>Technical details	SUBTYPES [Ref.]<br>Overall results
**CVCs**	**GENERIC CVCs [25, 27]**<br>**Surgical details**<br>• Percutaneous insertion:<br>○ Subclavian vein accessed infraclavicularly; local anesthesia; catheter (polyethylene or silicone) inserted, flushed, sutured, and dressed; chest X-ray confirms placement; maintenance includes antiseptic dressing changes and daily tubing replacement.<br>• Cutdown insertion:<br>○ Exposition of superficial vein; creation of subcutaneous tunnel; the silastic catheter is advanced with its Dacron cuff placed within the tunnel; after venous insertion, catheter is secured, flushed with heparin, and dressed; chest X-ray or fluoroscopy confirms positioning.<br>**Postoperative management**<br>• Frequent tubing and dressing changes required to reduce the risk of infection.	**GENERIC CVCs [25, 27]**<br>• Associated with higher infection rates, occlusion risks, early central venous stenosis, and increased mortality compared to AVGs.<br>• Require meticulous maintenance protocols to minimize complications.<br>• Better tolerated when totally implanted rather than externalized, offering improved patient comfort and ease of use.<br>• Indicated only in select clinical scenarios (e.g., pediatric patients <20 kg, severe cardiac dysfunction, terminal cancer), where AVFs or AVGs are unfeasible.<br>• Both studies emphasize the importance of individualized access planning to optimize long-term outcomes and reduce adverse events.
**AVFs**	**SIDE-TO-SIDE [11]**<br>**Surgical details**<br>• Local anesthesia.<br>• 5cm longitudinal incision at the wrist to expose the radial artery and cephalic vein.<br>• Side-to-side anastomosis is created using 5-0 Prolene suture.<br>• Average operative time was 30–35 minutes.<br>**Postoperative management**<br>• Prophylactic antibiotics and NSAID administered for 5 days.<br>• Dressing removed on postoperative day 3; sutures removed on day 8.<br><br>**END-TO-SIDE [10, 21]**<br>**Rationale**<br>• Wrist AVF preferred as first-line approach.<br>• Elbow AVFs (or even autologous AVGs) were used in cases of compromised wrist vasculature.<br>**Surgical details**<br>• End-to-side AVF created under local anesthesia, at wrist (radial artery–cephalic vein) or elbow (brachial artery–cubital vein).<br>• Choice of site tailored to vascular availability<br>**Postoperative management**<br>• Universal anticoagulation except in thrombocytopenic patients (e.g., leukemia).<br>• Patients with a granulocyte count of less than 500/mm^3 were given oral antibiotics before surgery.<br>• Vascular access monitored postoperatively; re-exploration and thrombectomy performed in early failures.<br><br>**LOOP [22]**<br>**Surgical details**<br>• Cephalic vein looped and anastomosed end-to-side with the brachial artery in the arm.<br>• Saphenous vein looped and anastomosed end-to-side with the femoral artery in the leg.<br>• Sutures with 7-0 nonabsorbable stitches.<br>**Postoperative management**<br>• Bed rest for 5 days; access utilized after 2 weeks.	**SIDE-TO-SIDE [11]**<br>• 10 AVFs in CT-naïve patients to enhance forearm venous accessibility for peripheral CT infusion.<br>• 1-year failure rate of only 20%.<br>• Significant reduction in required venipunctures (mean 1.0 vs. 2.7 in historical controls).<br>• No adverse events reported.<br>• Highlighted minimal hospital resource utilization and improved patient comfort.<br><br>**END-TO-SIDE [10, 21]**<br>• Wrist AVFs showed a 64% success rate, while elbow AVFs 59%.<br>• In Bell's series, all AVFs failed within 48 hours due to early thrombosis from CT-induced venous damage. Autopsy revealed extensive fibrotic changes and venous strictures.<br>• Authors recommend early AVF creation before venous compromise to improve outcomes.<br>• Despite the thrombosis risk in cancer patients, AVFs may offer viable long-term access in selected cases.<br><br>**LOOP [22]**<br>• The loop AVF in the leg of pediatric patients was the operation of choice, adopted in 46 cases against only 3 loop AVF crafted in the arm.<br>• Only 2 out of 46 leg surgeries failed to obtain usable access.<br>• 4 cases of reported total failure with leg loop AVFs, plus 2 other cases which needed early thrombectomies but were eventually saved.<br>• In one case, ligation was needed because the fistula was contributing to cardiac failure following CT.<br>• 4 other long term failure reported.<br>• Only minor ad temporary adverse events noted, as edema and hematoma formation.

	• No antibiotic or anticoagulant prophylaxis reported.<br><br>**THOMAS SHUNTS [22]**<br>**Surgical details**<br>• Performed between the superficial femoral artery and the femoral vein.<br>• Used when immediate access is needed in patients with severely compromised upper limbs vasculature.<br>**Postoperative management**<br>• Not specified.	**THOMAS SHUNTS [22]**<br>• 16 shunts implanted; 13 functioned adequately throughout the treatment period.<br>• Reported complications included 2 thromboses and 1 infection.<br>• Considered a satisfactory alternative when arm access is unfeasible.
**Biologic AVGs**	**AUTOGRAFT [10]**<br>**Rationale**<br>• Radiocephalic AVF was the preferred technique.<br>• Elbow fistula adopted in case of exhausted cephalic vein or when the former radiocephalic one fails.<br>• If the whole arm vasculature was compromised, then LSV autotransplant is pursued.<br>**Surgical details**<br>• LSV was harvested and used as a tunneled AVG, placed in the inguinal region under general anesthesia.<br>**Postoperative management**<br>• Anticoagulation administered in all patients, except those with thrombocytopenia due to leukemia.<br>• Patients with a granulocyte count of less than 500/mm^3 were given oral antibiotics before surgery.<br><br>**ALLOGRAFT [22]**<br>**Rationale**<br>• Used in only 2 cases, where both arms and legs were unsuitable for vascular access.<br>**Surgical details**<br>• A preserved human umbilical vein was employed as an arteriovenous conduit, anastomosed between the femoral artery and vein.<br>**Postoperative management**<br>• Not specified.<br><br>**BOVINE [22, 23]**<br>**Rationale**<br>• Adopted in patients with exhausted or inadequate upper limb vasculature.<br>• Aimed to provide durable vascular access for repeated infusion therapies.<br>**Surgical details**<br>• Commercial bovine carotid artery grafts (e.g., Surgicos, J&J) implanted as upper or lower limb AVGs.<br>• Anastomoses performed end-to-side to brachial artery and vein, using monofilament polypropylene sutures.<br>• Subcutaneous tunneling employed in all cases.<br>• Anesthesia varied: local infiltration, regional block, or general, based on clinical status.<br>• Intraoperative heparinization administered; preoperative antibiotics and transfusions given when required.<br>**Postoperative management**<br>• Bed rest for 5 days post-implantation, followed by ambulation.	**AUTOGRAFT [10]**<br>• Radiocephalic AVF obtained a 64% success rate, while elbow AVF 59% and autologous AVG 77%.<br>• Early thrombosis identified as the most frequent complication despite anticoagulant use.<br>• Autologous AVGs outperformed PTFE ones, which were mentioned as unsuccessful in attempted forearm placements.<br>• The authors underlined the high risk of thrombosis in cancer patients but still concluded that AVFs and autologous AVGs may provide effective long-term vascular access in select cases.<br><br>**ALLOGRAFT [22]**<br>• Only available information presented is a reported trend toward edema formation.<br><br>**BOVINE [22, 23, 27]**<br>• In Levey study [22], Bovine AVGs were used in a minority of cases (5 out of 77); no specific complications (nor results) reported.<br>• Costantino [23] reported a mean graft patency of 4.5 months (5.8 months excluding early deaths); 81% remained functional at endpoint.<br>• Thrombosis was the leading complication (16 events in 12 patients), with a 44% salvage rate; infections occurred in 4 patients, controlled in only 2.<br>• Reintervention required in 35% of cases; no procedure-related deaths.<br>• High user acceptance and consistent performance noted.<br>• Cifarelli's review [27] highlighted good histologic compatibility and patency for bovine-derived grafts, but also lower puncture resistance and aneurysm risk.

	• Antibiotic prophylaxis maintained for 48–72 hours.<br>• Grafts matured for 3 weeks prior to initial use.<br>Vascular access performed by nursing staff with heparin lock or butterfly catheters, promptly removed after use.	
**Synthetic AVGs**	**PTFE [24-27]**<br>**Surgical details**<br>• Anesthesia used<br>o Local anesthesia employed in most cases, especially for upper-limb graft placement.<br>o General anesthesia used in specific situations, including lower-limb placement or pediatric patients.<br>• Preferred setup was upper limb placement and straight configuration, but lower limb and loop configuration was also used.<br>• During surgery, grafts were subcutaneously tunneled, usually flushed with heparinized saline and then anastomosed to the given vessels.<br>• Finally, antibiotic irrigation was applied in the incisional area, subcutaneous and subcuticular sutures were applied, and steri-strips and sterile gauze were used to cover the wound.<br>• The whole procedure usually took between 1 hour and half and 2 hours.<br>**Postoperative management**<br>• Early use for CT administration generally after 1 week of healing.<br>• Antithrombotic or antibiotic prophylaxis was not deemed as mandatory.<br><br>**DACRON [21, 24, 25]**<br>(Same as PTFE)	**PTFE [24-27]**<br>• Widely adopted synthetic option for long-term vascular access, preferred over Dacron for comparable outcomes and lower cost.<br>• Demonstrated acceptable long-term patency with variable complication rates: early and late thrombosis, rare infections, and up to 43% overall complication rate (with both maintained and loss of function of the graft).<br>• Function often preserved over than one year, with successful revision in selected cases.<br>• Straight configuration outperformed loop setup.<br>• Despite reported association with intimal hyperplasia at the venous anastomosis, PTFE AVGs remain favored over CVCs for their lower maintenance and infection rates and comparable patency.<br><br>**DACRON [21, 24, 25]**<br>• Dacron AVGs were sporadically employed, typically as an alternative to PTFE, with no evidence of superior performance.<br>• PTFE was consistently preferred due to similar efficacy and reduced cost.<br>• Reported clinical use was limited and outcomes rarely stratified by graft material.<br>• No significant data supporting Dacron's routine use in oncology settings.
**Bioengineered AVGs**	**NUDE [12]**<br>**Production methods**<br>• Electrospinning of PBS.<br>**Pursued analyses**<br>• Scaffold properties were evaluated through morphological, mechanical, and cytocompatibility assays with human dermal fibroblasts.<br>• Degradation assessed under simulated physiological conditions.<br><br>**FUNCTIONALIZED [28, 29]**<br>**Production methods**<br>• Bertanha et al. [28]<br>o Vena cava harvested from adult rabbits and decellularized using 0.1% SDS.<br>o Functionalized with autologous adipose MSCs and human PL supernatant.<br>• Li et al. [29]<br>o Electrospun PLLA/gelatin scaffold incorporating PRP-gel supernatant.<br>o Seeded with VECs and VSMCs.<br>**Pursued analyses**<br>• Bertanha et al. [28]<br>o Histological and immunohistochemical evaluation, along with ELISA tests.<br>• Li [29]<br>o Various cytological, histological and mechanical analyses conducted *in vitro*, along with ELISA tests and SEM imaging.<br>o *In vivo* implantation and anastomosis with the neck vasculature of adult male New Zealand white rabbits.	**NUDE [12]**<br>• Developed electrospun PBS-based tubular scaffolds (3.62 mm inner diameter) mimicking extracellular matrix.<br>• *In vitro* analyses demonstrated:<br>o Excellent mechanical strength, including suture retention, resistance to burst pressure, and elastic compliance.<br>o Controlled biodegradation profile, preserving scaffold integrity under physiological conditions.<br>o Cytocompatibility, with successful cell adhesion and proliferation on scaffold surfaces.<br><br>**FUNCTIONALIZED [28, 29]**<br>• Bertanha et al. [28]<br>o Enhanced endothelial differentiation in MSCs exposed to EIGFs, evidenced by elevated von Willebrand factor expression.<br>o Sustained Fascin protein levels, indicative of proper endothelial phenotype and cytoskeletal structure.<br>o Morphological features consistent with functional endothelial integration.<br>• Li et al. [29]<br>o No reported complications after 4-week follow-up.<br>o Improved cell infiltration, endothelialization, contractile function, neovascularization and reduced inflammatory response in PRP-coated grafts vs. controls.<br>o Only partial satisfactory mechanical results.

Document S1 – *Eligibility criteria and Report of research*

Eligibility criteria

Criteria 1 = not regarding the specific PICO
Criteria 2 = retracted
Criteria 3 = not ENG / not ITA
Criteria 4 = not accessible
Inserted = ok

Report of research

Research 1 – PDs for vascular reconstruction

PICO

- *Patient* = not specified
- ***Intervention* = only PDs for vascular reconstruction**
- ***Comparator* = any classical vascular access**
- *Outcome* = not specified

***PubMed* string**
("Platelet-Rich Plasma"[Mesh] OR Platelet Lysate) AND (Tissue Regeneration OR Vascular Regeneration OR Vascular Reconstruction) AND (((Venous OR Arterial OR Vascular) AND (Scaffold OR Graft OR Prosthesis)) OR (Arteriovenous Fistula OR AVF OR Bridge Fistula) OR Venous Access Device OR Central Venous Catheter OR (Peripherally Inserted Central Catheter OR PICC) OR (Catheterization OR Administration OR Injections OR Infusions) OR "Vascular Surgical Procedures"[Mesh])

***PubMed* access date**
10th of January 2025

***PubMed* results (889)**

1. 16816947
2. 17036988
3. 17224034
4. 17236215
5. 17374962
6. 17376715
7. 17383488
8. 17518588
9. 17615992
10. 17635045
11. 17884521
12. 17888881
13. 18028949
14. 18067601
15. 18159914
16. 18181148
17. 18204746
18. 18216669
19. 18318807
20. 18332761
21. 18365669
22. 18485475
23. 18523898
24. 18607174
25. 18651114
26. 18672703
27. 18685050
28. 18823276
29. 19014150
30. 19068594
31. 19155630
32. 19159413
33. 19205700
34. 19231923
35. 19333802
36. 19346267
37. 19380129
38. 19392780
39. 19402740
40. 19706229
41. 19711008
42. 19816290
43. 19825710
44. 19838676
45. 19896041
46. 19912722
47. 19924600
48. 20141991
49. 20160631
50. 20187900
51. 20223574
52. 20302447
53. 20362825
54. 20383304
55. 20458234
56. 20480789
57. 20491533
58. 20493521
59. 20508516
60. 20626759
61. 20682414
62. 20708345
63. 20708988
64. 20740273
65. 20810068
66. 20814217
67. 20875101
68. 20885230
69. 20920431
70. 20971543
71. 20973348
72. 21029038
73. 21039222
74. 21048180
75. 21074782
76. 21082164
77. 21082421
78. 21099156
79. 21106774
80. 21150152
81. 21158495
82. 21159991
83. 21175288
84. 21195687
85. 21196289
86. 21200169
87. 21238815
88. 21238844
89. 21281261
90. 21290856
91. 21406450
92. 21478245
93. 21492303
94. 21632978
95. 21635618
96. 21644923
97. 21678642
98. 21732159
99. 21735776
100. 21740368
101. 21740370
102. 21740371
103. 21740378
104. 21764073
105. 21822108
106. 21862277
107. 21980023
108. 21993865
109. 22006233
110. 22030945
111. 22150696
112. 22179187
113. 22210730
114. 22265335
115. 22270740

116. 22277992
117. 22349290
118. 22349601
119. 22438198
120. 22452340
121. 22456358
122. 22506358
123. 22525416
124. 22568331
125. 22621774
126. 22632688
127. 22647081
128. 22681647
129. 22697064
130. 22698936
131. 22733706
132. 22739982
133. 22771087
134. 22793069
135. 22796828
136. 22823336
137. 22835570
138. 22849574
139. 22863146
140. 22884637
141. 22885836
142. 22894643
143. 22906160
144. 22927147
145. 22951762
146. 22972393
147. 22976648
148. 22981779
149. 22998040
150. 23012935
151. 23031161
152. 23032594
153. 23097413
154. 23135872
155. 23141203
156. 23150187
157. 23155361
158. 23197780
159. 23197813
160. 23207329
161. 23230828
162. 23289869
163. 23306279
164. 23316648
165. 23329397
166. 23389788
167. 23403167
168. 23422785
169. 23427847
170. 23472861
171. 23489697
172. 23508047
173. 23519544
174. 23563814
175. 23586320
176. 23621096
177. 23627562
178. 23629979
179. 23660186
180. 23702498
181. 23710476
182. 23714970
183. 23726109
184. 23765126
185. 23779152
186. 23780535
187. 23830317
188. 23848423
189. 23885779
190. 23915433
191. 23924315
192. 23933379
193. 24047553
194. 24066341
195. 24080566
196. 24168291
197. 24212366
198. 24212369
199. 24212371
200. 24287209
201. 24325905
202. 24354616
203. 24362291
204. 24368097
205. 24374699
206. 24374700
207. 24379006
208. 24406591
209. 24431249
210. 24443021
211. 24469887
212. 24519184
213. 24563864
214. 24576548
215. 24630241
216. 24656523
217. 24712305
218. 24732653
219. 24857481
220. 24883322
221. 24888224
222. 24890049
223. 24970005
224. 24978937
225. 24995618
226. 25006854
227. 25012741
228. 25027676
229. 25028112
230. 25056987
231. 25065381
232. 25089566
233. 25120225
234. 25146020
235. 25150331
236. 25165699
237. 25176059
238. 25181726
239. 25184132
240. 25186188
241. 25193888
242. 25234121
243. 25250325
244. 25284020
245. 25287591
246. 25295767
247. 25369448
248. 25377191
249. 25440421
250. 25459571
251. 25473209
252. 25492574
253. 25562162
254. 25572379
255. 25583629
256. 25625433
257. 25643855
258. 25662492
259. 25670657
260. 25710554
261. 25728718
262. 25795623
263. 25801293
264. 25804324
265. 25813869
266. 25819103
267. 25823672
268. 25855914
269. 25864463
270. 25879032
271. 25886527
272. 25920633
273. 25968005
274. 25975971
275. 26017402
276. 26017592
277. 26048672
278. 26064903
279. 26075269
280. 26076897
281. 26081268
282. 26236743
283. 26325015
284. 26335660
285. 26400925
286. 26461101
287. 26481592
288. 26498958
289. 26614728
290. 26663430
291. 26689714
292. 26703066
293. 26748836
294. 26768536
295. 26832807
296. 26876351
297. 26879294
298. 26881220
299. 26910664
300. 26923362
301. 26924405
302. 26927211
303. 26956080
304. 26961805
305. 26967070
306. 26980293
307. 26981170
308. 26985986
309. 27015236
310. 27043959
311. 27051343
312. 27063081
313. 27071140
314. 27109909
315. 27113287
316. 27119924
317. 27125598
318. 27141997
319. 27161868
320. 27191987
321. 27217525
322. 27222038
323. 27255146
324. 27255378
325. 27258008
326. 27310019
327. 27324079
328. 27346565
329. 27347938
330. 27400716
331. 27443002
332. 27474688
333. 27475686
334. 27481642
335. 27535268
336. 27551718
337. 27552452
338. 27567559
339. 27609047
340. 27638154
341. 27665095
342. 27677916
343. 27695328
344. 27706690
345. 27712135
346. 27817884
347. 27837344
348. 27845852
349. 27914126
350. 27943660
351. 27955807
352. 28027872
353. 28084000
354. 28097149
355. 28127660
356. 28127950
357. 28154995
358. 28161837
359. 28165420
360. 28175965
361. 28191592
362. 28194878
363. 28202370
364. 28236164
365. 28246352
366. 28249808
367. 28251260
368. 28269759
369. 28283682
370. 28291948
371. 28296878
372. 28301205
373. 28315407
374. 28339434
375. 28351189
376. 28452719
377. 28453295
378. 28464195
379. 28501713
380. 28504480
381. 28509340
382. 28538802
383. 28545565
384. 28576462
385. 28635382
386. 28672135
387. 28673602
388. 28688621
389. 28760125
390. 28782384
391. 28823090
392. 28834840
393. 28846786
394. 28853968
395. 28902937
396. 28915522
397. 29047244
398. 29049258
399. 29058626
400. 29076290
401. 29161717
402. 29209937
403. 29211969
404. 29226630
405. 29254291
406. 29266290
407. 29270414
408. 29316815
409. 29465055
410. 29483815
411. 29486615
412. 29503013
413. 29507631
414. 29510094
415. 29522657
416. 29536393
417. 29564054
418. 29574015
419. 29577749
420. 29604139
421. 29608156
422. 29663706
423. 29682870
424. 29715071
425. 29730840
426. 29736577
427. 29766749
428. 29847961
429. 29868646
430. 29890349
431. 29964256
432. 29978342
433. 29981946
434. 29994865
435. 30031163
436. 30044706
437. 30092110
438. 30102625
439. 30104096
440. 30110184
441. 30132050
442. 30150580
443. 30174103
444. 30195260
445. 30208342
446. 30232343
447. 30259059
448. 30259137
449. 30272521
450. 30306686

451. 30306698
452. 30348699
453. 30366137
454. 30370433
455. 30388866
456. 30420268
457. 30440099
458. 30460739
459. 30481239
460. 30500725
461. 30527473
462. 30579747
463. 30585053
464. 30587605
465. 30606055
466. 30628121
467. 30658950
468. 30678399
469. 30688814
470. 30699495
471. 30706995
472. 30725347
473. 30742878
474. 30758709
475. 30767566
476. 30809666
477. 30841510
478. 30855461
479. 30894109
480. 30898594
481. 30912141
482. 30913049
483. 30915890
484. 30919033
485. 30929771
486. 30937491
487. 30964227
488. 31008759
489. 31010247
490. 31013535
491. 31037585
492. 31038850
493. 31070298
494. 31074457
495. 31081004
496. 31082278
497. 31100937
498. 31126348
499. 31144021
500. 31144785
501. 31148358
502. 31150275
503. 31172647
504. 31180258
505. 31223618
506. 31227435
507. 31241854
508. 31269114
509. 31269346
510. 31280539
511. 31287918
512. 31307095
513. 31320230
514. 31326128
515. 31327129
516. 31344305
517. 31414709
518. 31428042
519. 31445759
520. 31448554
521. 31449340
522. 31453904
523. 31471902
524. 31476140
525. 31481694
526. 31489852
527. 31516089
528. 31531371
529. 31560791
530. 31594187
531. 31600109
532. 31626238
533. 31658332
534. 31665898
535. 31739890
536. 31785081
537. 31788748
538. 31789622
539. 31802614
540. 31846610
541. 31852618
542. 31867928
543. 31971046
544. 32045103
545. 32051476
546. 32061047
547. 32061058
548. 32108322
549. 32131655
550. 32144565
551. 32219975
552. 32251339
553. 32285617
554. 32286865
555. 32312142
556. 32322459
557. 32333512
558. 32339370
559. 32341700
560. 32366164
561. 32370144
562. 32376297
563. 32382929
564. 32424462
565. 32503405
566. 32514742
567. 32519886
568. 32530334
569. 32538130
570. 32540361
571. 32540414
572. 32543878
573. 32567485
574. 32591195
575. 32597779
576. 32608166
577. 32638844
578. 32643473
579. 32658438
580. 32668325
581. 32700986
582. 32718036
583. 32748436
584. 32766312
585. 32770685
586. 32820598
587. 32827074
588. 32859524
589. 32888384
590. 32897011
591. 32937996
592. 32962283
593. 32962673
594. 33036225
595. 33049123
596. 33068206
597. 33078204
598. 33232565
599. 33258707
600. 33299533
601. 33308245
602. 33331973
603. 33361845
604. 33382002
605. 33401977
606. 33429694
607. 33429777
608. 33433526
609. 33441277
610. 33446439
611. 33462983
612. 33484919
613. 33546534
614. 33559114
615. 33559623
616. 33596441
617. 33597001
618. 33628814
619. 33632047
620. 33649927
621. 33650437
622. 33691459
623. 33712331
624. 33740995
625. 33745654
626. 33781654
627. 33791377
628. 33791879
629. 33833044
630. 33838005
631. 33845642
632. 33850460
633. 33880860
634. 33970255
635. 33982442
636. 34002724
637. 34008330
638. 34021518
639. 34044843
640. 34060090
641. 34072505
642. 34086871
643. 34090286
644. 34106813
645. 34169656
646. 34218061
647. 34228901
648. 34287259
649. 34288258
650. 34293441
651. 34318612
652. 34373751
653. 34384663
654. 34415119
655. 34481617
656. 34508762
657. 34524548
658. 34549620
659. 34558456
660. 34581513
661. 34704498
662. 34710335
663. 34715152
664. 34718850
665. 34807418
666. 34814693
667. 34816309
668. 34847111
669. 34861782
670. 34870370
671. 34873572
672. 34885481
673. 34896242
674. 34918648
675. 34973742
676. 35051139
677. 35070865
678. 35094131
679. 35108667
680. 35124154
681. 35152730
682. 35216052
683. 35234122
684. 35271553
685. 35291806
686. 35297377
687. 35328437
688. 35352743
689. 35377044
690. 35395489
691. 35430904
692. 35435027
693. 35435087
694. 35462516
695. 35487496
696. 35527147
697. 35538825
698. 35633608
699. 35659167
700. 35661102
701. 35725198
702. 35750991
703. 35791055
704. 35805907
705. 35841966
706. 35904836
707. 35975804
708. 36056770
709. 36059533
710. 36098849
711. 36101858
712. 36176925
713. 36181298
714. 36219518
715. 36219860
716. 36242639
717. 36265834
718. 36274217
719. 36290482
720. 36318219
721. 36329527
722. 36330669
723. 36346560
724. 36361627
725. 36371198
726. 36395946
727. 36401577
728. 36403073
729. 36403416
730. 36410882
731. 36496199
732. 36520210
733. 36520838
734. 36546662
735. 36573287
736. 36606396
737. 36669628
738. 36675025
739. 36676735
740. 36684076
741. 36688861
742. 36721958
743. 36742986
744. 36760757
745. 36762408
746. 36768532
747. 36769065
748. 36801280
749. 36864471
750. 36881139
751. 36894793
752. 36907047
753. 36913891
754. 37002441
755. 37039525
756. 37047135
757. 37073922
758. 37118335
759. 37125516
760. 37162458
761. 37171916
762. 37191915
763. 37230636
764. 37354139
765. 37354612
766. 37365609
767. 37400671
768. 37409489
769. 37418583
770. 37420147
771. 37438668
772. 37438670
773. 37443831
774. 37468823
775. 37469259
776. 37501011
777. 37515478
778. 37528751
779. 37580431
780. 37584506
781. 37606714
782. 37620826
783. 37666466
784. 37668753
785. 37702275

786. 37721981
787. 37726689
788. 37758244
789. 37763186
790. 37805683
791. 37805801
792. 37834357
793. 37939238
794. 37941067
795. 37986559
796. 38003297
797. 38037213
798. 38040732
799. 38048184
800. 38058166
801. 38085349
802. 38093400
803. 38114998
804. 38153510
805. 38196007
806. 38197356
807. 38200124
808. 38227215
809. 38229261
810. 38280707
811. 38306618
812. 38316648
813. 38334783
814. 38371711
815. 38422207
816. 38424182
817. 38430019
818. 38457479
819. 38518234
820. 38536994
821. 38558364
822. 38568824
823. 38654547
824. 38655988
825. 38674274
826. 38678608
827. 38701430
828. 38710570
829. 38754675
830. 38759486
831. 38782801
832. 38786069
833. 38806836
834. 38823938
835. 38831361
836. 38851675
837. 38867383
838. 38909628
839. 38951079
840. 38964528
841. 38967341
842. 38976297
843. 39001964
844. 39020391
845. 39074896
846. 39110247
847. 39115162
848. 39145857
849. 39158761
850. 39195210
851. 39215319
852. 39230578
853. 39237980
854. 39244111
855. 39246090
856. 39262041
857. 39280316
858. 39299368
859. 39307596
860. 39329304
861. 39358919
862. 39369014
863. 39369241
864. 39426253
865. 39438767
866. 39472866
867. 39476786
868. 39495461
869. 39498821
870. 39519084
871. 39531857
872. 39533589
873. 39539934
874. 39543829
875. 39597082
876. 39610694
877. 39630665
878. 39655847
879. 39668631
880. 39673358
881. 39702943
882. 39715139
883. 39721047
884. 39740095
885. 39755875
886. 39799024
887. 39822342
888. 40213674
889. 40386186

***PubMed* screening**

Criteria 1 = 881
Criteria 2 = 3
Criteria 3 = 3
Criteria 4 = 0
Inserted = 2

***Cochrane Library* string**

ID	Search	Hits
**#1**	Platelet-Rich Plasma	3837
**#2**	Platelet Lysate	82
**#3**	Tissue Regeneration	3128
**#4**	Vascular Regeneration	401
**#5**	Vascular Reconstruction	713
**#6**	Venous	36175
**#7**	Arterial	64384
**#8**	Vascular	67320
**#9**	Scaffold	1078
**#10**	Graft	33503
**#11**	Prosthesis	16617
**#12**	Arteriovenous Fistula	1607
**#13**	AVF	754
**#14**	Bridge Fistula	39
**#15**	Venous Access Device	844
**#16**	Central Venous Catheter	5132
**#17**	Peripherally Inserted Central Catheter	560
**#18**	PICC	596
**#19**	Catheterization	15116
**#20**	Administration	435962
**#21**	Injections	52709
**#22**	Infusions	24919
**#23**	Vascular Surgical Procedures	3762
**#24**	#1 OR #2	3901
**#25**	#3 OR #4 OR #5	3989
**#26**	#6 OR #7 OR #8	141708
**#27**	#9 OR #10 OR #11	49598
**#28**	#26 AND #27	7734
**#29**	#12 OR #13 OR #14	1830
**#30**	#17 OR #18	778
**#31**	#19 OR #20 OR #21 OR #22	473197
**#32**	#28 OR #29 OR #15 OR #16 OR #30 OR #31	482386

#33	#24 AND #25 AND #32	116

***Cochrane Library* access date**
14th of January 2025

***Cochrane Library* results (116)**

1. CD005511.PUB3
2. CD005968.PUB3
3. CD008455.PUB2
4. CD009461.PUB4
5. CD009496.PUB2
6. CD009768.PUB2
7. CD010071.PUB3
8. CD010764.PUB2
9. CD010951.PUB2
10. CD011423.PUB2
11. CD011990.PUB2
12. CD012128.PUB2
13. CD012432.PUB3
14. CD013332.PUB2
15. CD013341
16. CD013342
17. CD013439.PUB2
18. CD013875.PUB2
19. CD014328
20. CD014918.PUB2
21. CN-00378508
22. CN-00502549
23. CN-00513445
24. CN-00812354
25. CN-00875955
26. CN-00892141
27. CN-00917801
28. CN-00961457
29. CN-00963719
30. CN-00967120
31. CN-01011851
32. CN-01336795
33. CN-01369989
34. CN-01441730
35. CN-01475901
36. CN-01493634
37. CN-01533607
38. CN-01547050
39. CN-01551396
40. CN-01560430
41. CN-01561363
42. CN-01565353
43. CN-01567406
44. CN-01594042
45. CN-01613260
46. CN-01654913
47. CN-01656459
48. CN-01660319
49. CN-01660894
50. CN-01661851
51. CN-01713334
52. CN-01745567
53. CN-01777126
54. CN-01796178
55. CN-01845017
56. CN-01915084
57. CN-01919052
58. CN-01941749
59. CN-01969498
60. CN-01981146
61. CN-01985856
62. CN-01985863
63. CN-01990425
64. CN-02077977
65. CN-02090718
66. CN-02103259
67. CN-02136868
68. CN-02172427
69. CN-02175102
70. CN-02193132
71. CN-02195768
72. CN-02206053
73. CN-02264561
74. CN-02282687
75. CN-02289361
76. CN-02322784
77. CN-02326119
78. CN-02347180
79. CN-02352975
80. CN-02353865
81. CN-02359861
82. CN-02394983
83. CN-02431039
84. CN-02440383
85. CN-02452061
86. CN-02474661
87. CN-02488126
88. CN-02498446
89. CN-02508917
90. CN-02509799
91. CN-02511424
92. CN-02517102
93. CN-02531000
94. CN-02539732
95. CN-02543810
96. CN-02558899
97. CN-02559698
98. CN-02562634
99. CN-02564033
100. CN-02566871
101. CN-02572091
102. CN-02575469
103. CN-02606127
104. CN-02617737
105. CN-02651918
106. CN-02666139
107. CN-02676116
108. CN-02682766
109. CN-02698387
110. CN-02698427
111. CN-02741879
112. CN-02756570
113. CN-02766680
114. CN-02773237
115. CN-02780464
116. CN-02791378

***Cochrane Library* screening**
Criteria 1 = 116
Criteria 2 = 0
Criteria 3 = 0
Criteria 4 = 0
Inserted = 0

Research 2 – PBS for vascular reconstruction

PICO

- *Patient* = not specified
- ***Intervention* = only PRP for vascular reconstruction**
- ***Comparator* = any classical vascular access**
- *Outcome* = not specified

***PubMed* string**
("Platelet-Rich Plasma"[Mesh] OR Platelet Lysate) AND (Tissue Regeneration OR Vascular Regeneration OR Vascular Reconstruction) AND (((Venous OR Arterial OR Vascular) AND (Scaffold OR Graft OR Prosthesis)) OR (Arteriovenous Fistula OR AVF OR Bridge Fistula) OR Venous Access Device OR Central Venous Catheter OR (Peripherally Inserted Central Catheter OR PICC) OR (Catheterization OR Administration OR Injections OR Infusions) OR "Vascular Surgical Procedures"[Mesh])

***PubMed* access date**
10th of January 2025

***PubMed* results (392)**

1. 1975615
2. 2301991
3. 2450779
4. 3607481
5. 7487375
6. 7683531
7. 7687087
8. 8613731
9. 9337553
10. 9337558
11. 9875263
12. 9893600
13. 10608742
14. 10632395
15. 10966842
16. 10976751
17. 11523246
18. 11733085
19. 11813541
20. 12022744
21. 12579151
22. 12890377
23. 12932714
24. 14579933
25. 14651811
26. 14657916
27. 14974963
28. 15128098
29. 15145210
30. 15164170
31. 15231019
32. 15282143
33. 15322216
34. 15522759
35. 15648736
36. 15883211
37. 16049978
38. 16245268
39. 16392139
40. 16461370
41. 16557552
42. 16859799
43. 17066631
44. 17330875
45. 17551103
46. 17643267
47. 17644111
48. 17950455
49. 18159003
50. 18310229
51. 18364144
52. 18541296
53. 18555991
54. 18556121
55. 18600489
56. 18691637
57. 18824741
58. 18824771
59. 18825159
60. 18845078
61. 18974384
62. 19031139
63. 19040421
64. 19137896
65. 19320744
66. 19478831
67. 19745189
68. 19885863
69. 19918893
70. 20030119
71. 20157290
72. 20209939
73. 20214792
74. 20404540
75. 20533597
76. 20659357
77. 20667614
78. 20669677
79. 20889618
80. 20937252
81. 20937527
82. 20948411
83. 21035182
84. 21135814
85. 21191126
86. 21197220
87. 21210502
88. 21254389
89. 21270241
90. 21344499
91. 21431359
92. 21538185
93. 21591793
94. 21632142
95. 21667244
96. 21697550
97. 21793325
98. 21838774
99. 21860575
100. 21889218
101. 21929873
102. 22061489
103. 22314040
104. 22443121
105. 22490536
106. 22570298
107. 22588929
108. 22669154
109. 22750747
110. 22828989
111. 23012935
112. 23103791
113. 23132806
114. 23182165
115. 23193024
116. 23201131
117. 23206704
118. 23220731
119. 23290003
120. 23303720
121. 23372696
122. 23428649
123. 23455045
124. 23473301
125. 23576927
126. 23595964
127. 23613940
128. 23665256
129. 23685144
130. 23805264
131. 23830814
132. 23892469
133. 24011427
134. 24039961
135. 24054847
136. 24075481
137. 24122925
138. 24204700
139. 24260375
140. 24295296
141. 24307236
142. 24488438
143. 24501171
144. 24589605
145. 24604133
146. 24643010
147. 24649870
148. 24963878
149. 24965023
150. 25099499
151. 25210842
152. 25253391
153. 25284727
154. 25433606
155. 25434870
156. 25545323
157. 25562162
158. 25647659
159. 25661977
160. 26052759
161. 26065900
162. 26096962
163. 26112755
164. 26185380
165. 26221480
166. 26294008
167. 26320084
168. 26330843
169. 26419946
170. 26428904
171. 26491459
172. 26562835
173. 26564465
174. 26577999
175. 26636416
176. 26750016
177. 26911882
178. 27007787
179. 27058215
180. 27211359
181. 27255234
182. 27281869
183. 27327351
184. 27413735
185. 27431811
186. 27494445
187. 27567556
188. 27600523
189. 27711227
190. 27770950
191. 27817105
192. 27825159
193. 27877076
194. 27987787
195. 28031116
196. 28135618
197. 28138705
198. 28420433
199. 28427476
200. 28439674
201. 28487969
202. 28539277
203. 28554003
204. 28615039
205. 28653257
206. 28660411
207. 28687961
208. 28704409
209. 28714022
210. 28778713
211. 28790053
212. 28869865
213. 28877384
214. 28938788
215. 28965080
216. 28983620
217. 29020441
218. 29091962
219. 29134575
220. 29176876
221. 29198777
222. 29240284
223. 29290803
224. 29408092
225. 29637382
226. 29786307
227. 29806371
228. 29845352
229. 29905053
230. 29997088
231. 30041250
232. 30214505
233. 30232729
234. 30250486
235. 30343040
236. 30345545
237. 30463592
238. 30504732
239. 30506493
240. 30528606
241. 30535488
242. 30541703
243. 30569689
244. 30635673
245. 30666428
246. 30678400
247. 30867059
248. 30879244
249. 30911550
250. 30963640
251. 31040645
252. 31113469
253. 31129807
254. 31187334
255. 31197995
256. 31215503
257. 31259836
258. 31327961
259. 31391117
260. 31456291
261. 31582220
262. 31633702
263. 31673085
264. 31809714
265. 31885617
266. 31939247
267. 31953639
268. 32030956
269. 32068240
270. 32078052
271. 32119015
272. 32154243
273. 32206735
274. 32241045
275. 32321586
276. 32377717
277. 32502570
278. 32545915
279. 32600714
280. 32619986
281. 32621158
282. 32676953
283. 32778240
284. 32787917
285. 32828592
286. 32848391
287. 32901495
288. 32924817
289. 32929341
290. 33061381
291. 33105942
292. 33455199
293. 33678130
294. 33716780
295. 33813603

296. 33864528	316. 35447848	336. 36754122	356. 37954299	376. 38962170
297. 33999216	317. 35450516	337. 36768255	357. 37965106	377. 39020406
298. 34200896	318. 35590084	338. 36878522	358. 37969023	378. 39034645
299. 34372877	319. 35599422	339. 36899900	359. 37977318	379. 39053176
300. 34541411	320. 35669002	340. 36932362	360. 38176487	380. 39073096
301. 34636236	321. 35802247	341. 36950151	361. 38296235	381. 39103918
302. 34645152	322. 35986406	342. 36970598	362. 38297365	382. 39142250
303. 34648803	323. 36076544	343. 37159455	363. 38302709	383. 39200128
304. 34655846	324. 36126655	344. 37173025	364. 38347646	384. 39227794
305. 34782616	325. 36242527	345. 37194321	365. 38350358	385. 39293850
306. 34815807	326. 36242919	346. 37296591	366. 38365799	386. 39300889
307. 34855833	327. 36453283	347. 37400586	367. 38386369	387. 39318262
308. 34904366	328. 36518468	348. 37421548	368. 38463959	388. 39365192
309. 34926699	329. 36551913	349. 37620943	369. 38592032	389. 39450249
310. 34955403	330. 36552813	350. 37650330	370. 38624019	390. 39491520
311. 35038115	331. 36593024	351. 37651346	371. 38664026	391. 39791406
312. 35265215	332. 36603371	352. 37659491	372. 38679747	392. 39981314
313. 35278880	333. 36624478	353. 37730257	373. 38804080	
314. 35294246	334. 36633616	354. 37842845	374. 38844606	
315. 35303271	335. 36740424	355. 37872294	375. 38859891	

***PubMed* screening**
Criteria 1 = 380
Criteria 2 = 3
Criteria 3 = 9
Criteria 4 = 0
Inserted = 0
Additional relevant paper = 1 → PMID 36365480

***Cochrane Library* string**

ID	Search	Hits
**#1**	Polybutylene Succinate	0
**#2**	PBS	1043
**#3**	Phosphate Buffered Saline	322
**#4**	Tissue Regeneration	3128
**#5**	Vascular Regeneration	401
**#6**	Vascular Reconstruction	713
**#7**	Venous	36175
**#8**	Arterial	64384
**#9**	Vascular	67320
**#10**	Scaffold	1078
**#11**	Graft	33503
**#12**	Prosthesis	16617
**#13**	Arteriovenous Fistula	1607
**#14**	AVF	754
**#15**	Bridge Fistula	39
**#16**	Venous Access Device	844
**#17**	Central Venous Catheter	5132
**#18**	Peripherally Inserted Central Catheter	560
**#19**	PICC	596
**#20**	Catheterization	15116
**#21**	Administration	435962
**#22**	Injections	52709
**#23**	Infusions	24919
**#24**	Vascular Surgical Procedures	3762
**#25**	#1 OR #2 NOT #3	908
**#26**	#4 OR #5 OR #6	3989
**#27**	#7 OR #8 OR #9	141708
**#28**	#10 OR #11 OR #12	49598
**#29**	#27 AND #28	7734
**#30**	#13 OR #14 OR #15	1830
**#31**	#18 OR #19	778
**#32**	#20 OR #21 OR #22 OR #23	473197

**#33**	#29 OR #30 OR #16 OR #17 OR #31 OR #32 OR #24	484140
**#34**	#25 AND #26 AND #33	1

***Cochrane Library* access date**
14th of January 2025

***Cochrane Library* results (1)**

1. CN-01713334

***Cochrane Library* screening**
Criteria 1 = 1
Criteria 2 = 0
Criteria 3 = 0
Criteria 4 = 0
Inserted = 0

Research 3 – PBS & PDs

PICO

- *Patient* = not specified
- ***Intervention* = any application of PBS + PDs**
- *Comparator* = not specified
- *Outcome* = not specified

***PubMed* string →** *("Platelet-Rich Plasma"[Mesh] OR Platelet Lysate) AND (Polybutylene Succinate OR PBS NOT Phosphate Buffered Saline)*

***PubMed* access date**
10th of January 2025

***PubMed* results (27)**

1. 1531707
2. 18001596
3. 20585536
4. 20695387
5. 21521235
6. 21889954
7. 23012935
8. 23898508
9. 24125774
10. 24368099
11. 25562162
12. 26353774
13. 26781753
14. 28763452
15. 28765945
16. 31034039
17. 31090348
18. 33224943
19. 34422211
20. 34656163
21. 35348136
22. 35936551
23. 36116591
24. 36542954
25. 36585527
26. 38189392
27. 38995815

***PubMed* screening**
Criteria 1 = 21
Criteria 2 = 1
Criteria 3 = 5
Criteria 4 = 0
Inserted = 0

***Cochrane Library* string**

ID	Search	Hits
**#1**	Platelet-Rich Plasma	3837
**#2**	Platelet Lysate	82
**#3**	Polybutylene Succinate	0
**#4**	PBS	1043
**#5**	Phosphate Buffered Saline	322

#6	#1 OR #2	3901
#7	#3 OR #4 NOT #5	908
#8	#6 AND #7	4

***Cochrane Library* access date**
14th of January 2025

***Cochrane Library* results (4)**

1. CN-01713334
2. CN-01954817
3. CN-02269581
4. CN-02499269

***Cochrane Library* screening**
Criteria 1 = 4
Criteria 2 = 0
Criteria 3 = 0
Criteria 4 = 0
Inserted = 0

Research 4 – Vascular graft or fistula for CT

PICO

- ***Patient* = undergoing chemotherapy**
- ***Intervention* = any graft or fistula for peripheral vascular access**
- ***Comparator* = any classical vascular access**
- *Outcome* = not specified

***PubMed* string**
("Neoplasms"[Mesh] OR "cancer"[tiab] OR "tumor*"[tiab] OR "tumour*"[tiab]) AND ((Venous OR Arterial OR Vascular) AND (Scaffold OR Graft OR Prosthesis)) AND ((Arteriovenous Fistula OR AVF OR Bridge Fistula) OR Venous Access Device OR Central Venous Catheter OR (Peripherally Inserted Central Catheter OR PICC))*

***PubMed* access date**
10th of January 2025

***PubMed* results (674)**

1. 109934
2. 273405
3. 290169
4. 338574
5. 451685
6. 507972
7. 569692
8. 580739
9. 648267
10. 775643
11. 849146
12. 1164079
13. 1423257
14. 1465287
15. 1490203
16. 1540103
17. 1571709
18. 1600417
19. 1676322
20. 1677913
21. 1772859
22. 1857858
23. 1859894
24. 1865699
25. 1886583
26. 1903693
27. 1913463
28. 1948571
29. 1968484
30. 1976016
31. 2068334
32. 2121346
33. 2149052
34. 2156415
35. 2161702
36. 2234258
37. 2239032
38. 2309152
39. 2334831
40. 2340466
41. 2376387
42. 2386917
43. 2404087
44. 2551229
45. 2597624
46. 2647187
47. 2665792
48. 2666137
49. 2716332
50. 2915237
51. 2918491
52. 2918492
53. 2986195
54. 3079946
55. 3107937
56. 3109299
57. 3126086
58. 3132566
59. 3155237
60. 3236954
61. 3358106
62. 3549932
63. 3551382
64. 3657878
65. 3721802
66. 3762187
67. 3777336
68. 3783829
69. 3822030
70. 3880834
71. 3886250
72. 3892837
73. 3918200
74. 3918783
75. 3936005
76. 3940846
77. 3943036
78. 4025917
79. 4037644
80. 4355359
81. 4548949
82. 4878669
83. 5013721
84. 6127750
85. 6344817
86. 6348395
87. 6349341
88. 6377003
89. 6382738
90. 6394757
91. 6453696
92. 6716582
93. 6766831
94. 6800275
95. 6883281
96. 7048043
97. 7123491
98. 7316044
99. 7449088
100. 7536025
101. 7552791
102. 7652839
103. 7742759
104. 7759829
105. 7763065

106. 7795246
107. 7807561
108. 7842070
109. 7866069
110. 7912601
111. 7933738
112. 8027806
113. 8037004
114. 8098313
115. 8156260
116. 8189590
117. 8284565
118. 8330503
119. 8365459
120. 8365461
121. 8385296
122. 8392831
123. 8397039
124. 8512970
125. 8582676
126. 8722358
127. 8722372
128. 8733712
129. 8766179
130. 8792352
131. 8992390
132. 9176771
133. 9180913
134. 9190299
135. 9200028
136. 9244921
137. 9252953
138. 9297394
139. 9346676
140. 9361054
141. 9367491
142. 9406362
143. 9407493
144. 9587869
145. 9617864
146. 9639627
147. 9645577
148. 9655009
149. 9669690
150. 9733276
151. 9737250
152. 9779064
153. 9779635
154. 9793013
155. 9828018
156. 9840035
157. 9848842
158. 10037049
159. 10082101
160. 10155708
161. 10171167
162. 10191709
163. 10227789
164. 10328158
165. 10370001
166. 10378205
167. 10397119
168. 10435741
169. 10440451
170. 10457333
171. 10475886
172. 10481576
173. 10505037
174. 10585136
175. 10664496
176. 10673672
177. 10673676
178. 10725979
179. 10745265
180. 10806626
181. 10830752
182. 10853751
183. 10868530
184. 10894140
185. 10976248
186. 10997820
187. 11013040
188. 11042661
189. 11044233
190. 11103081
191. 11111232
192. 11197219
193. 11204920
194. 11339151
195. 11340711
196. 11389302
197. 11428882
198. 11452403
199. 11469097
200. 11497393
201. 11680834
202. 11778073
203. 11781648
204. 11901015
205. 11904791
206. 11907745
207. 11907778
208. 11972243
209. 12136255
210. 12182427
211. 12183835
212. 12235524
213. 12352633
214. 12481455
215. 12515021
216. 12517836
217. 12522636
218. 12559022
219. 12607590
220. 12692629
221. 12695814
222. 12714226
223. 12717792
224. 12725356
225. 12735141
226. 12748671
227. 12772131
228. 12789478
229. 12883804
230. 12917144
231. 12942573
232. 14089095
233. 14158220
234. 14158221
235. 14170155
236. 14505156
237. 14612164
238. 14617015
239. 14676783
240. 14696451
241. 14700414
242. 14709689
243. 14746231
244. 14760125
245. 15080311
246. 15095217
247. 15178717
248. 15219065
249. 15227219
250. 15251387
251. 15266176
252. 15281527
253. 15334865
254. 15359641
255. 15365798
256. 15370671
257. 15386305
258. 15519483
259. 15621135
260. 15631921
261. 15636290
262. 15676137
263. 15686729
264. 15758877
265. 15789791
266. 15800767
267. 15849651
268. 15889215
269. 15909604
270. 15953009
271. 15997431
272. 16001935
273. 16029971
274. 16035098
275. 16115109
276. 16254671
277. **16258474**
278. 16288352
279. 16319054
280. 16358302
281. 16369969
282. 16377030
283. 16394888
284. 16420911
285. 16424972
286. 16427330
287. 16498035
288. 16498041
289. 16520947
290. 16596418
291. 16620623
292. 16622649
293. 16649737
294. 16679939
295. 16734883
296. 16845866
297. 16883607
298. 16892146
299. 16945048
300. 16956483
301. 16969041
302. 17023104
303. 17103072
304. 17112839
305. 17239129
306. 17310443
307. 17324762
308. 17337384
309. 17355999
310. 17369921
311. 17396089
312. 17506959
313. 17518180
314. 17533457
315. 17537488
316. 17540535
317. 17570192
318. 17605068
319. 17638264
320. 17723127
321. 17845155
322. 17888008
323. 17891851
324. 17976941
325. 18030555
326. 18202420
327. 18225574
328. 18303205
329. 18322718
330. 18342947
331. 18379981
332. 18446117
333. 18478498
334. 18621987
335. 18622420
336. 18623164
337. 18626247
338. 18656009
339. 18690596
340. 18716418
341. 18755652
342. 18797200
343. 18831907
344. 18992048
345. 19097806
346. 19139891
347. 19144528
348. 19231558
349. 19327941
350. 19339200
351. 19350170
352. 19376426
353. 19377926
354. 19382069
355. 19411734
356. 19453696
357. 19542067
358. 19550129
359. 19562624
360. 19604552
361. 19708941
362. 19722765
363. 19738109
364. 19816634
365. 19819688
366. 19827352
367. 19853734
368. 19921990
369. 19967773
370. 20004580
371. 20034943
372. 20127333
373. 20141056
374. 20169960
375. 20191610
376. 20204533
377. 20308910
378. 20348043
379. 20352161
380. 20523163
381. 20530452
382. 20534305
383. 20651636
384. 20678961
385. 20690530
386. 20958986
387. 20971550
388. 21125981
389. 21154387
390. 21186148
391. 21435763
392. 21479744
393. 21494530
394. 21554240
395. 21599752
396. 21621378
397. 21688246
398. 21915896
399. 21927566
400. 21947022
401. 21976039
402. 21994284
403. 21994880
404. 22011534
405. 22046874
406. 22176495
407. 22179804
408. 22270939
409. 22425356
410. 22459743
411. 22524597
412. 22531452
413. 22544224
414. 22550374
415. 22661169
416. 22698657
417. 22744260
418. 22864473
419. 22878577
420. 22882156
421. 22885191
422. 22951064
423. 23006521
424. 23040605
425. 23043572
426. 23090254
427. 23137137
428. 23238840
429. 23255071
430. 23380342
431. 23430671
432. 23601358
433. 23615918
434. 23725464
435. 23838215
436. 23848936
437. 23889746
438. 23890402
439. 23920294
440. 23938114

441. 23956157
442. 23996135
443. 24024476
444. 24118625
445. 24183597
446. 24185587
447. 24192771
448. 24263218
449. 24270071
450. 24315003
451. 24320593
452. 24405847
453. 24490064
454. 24550232
455. 24595880
456. 24618390
457. 24720144
458. 24797402
459. 24840744
460. 24842526
461. 24861690
462. 24902424
463. 25051279
464. 25053907
465. 25063013
466. 25072372
467. 25097705
468. 25108876
469. 25110299
470. 25264363
471. 25278398
472. 25317335
473. 25359689
474. 25362986
475. 25557252
476. 25632993
477. 25651878
478. 25671769
479. 25680325
480. 25804192
481. 25808822
482. 25851934
483. 25911604
484. 25912636
485. 25912774
486. 25913035
487. 25923973
488. 25953513
489. 26057184
490. 26119639
491. 26201750
492. 26315562
493. 26316482
494. 26322512
495. 26361623
496. 26507141
497. 26516306
498. 26662292
499. 26750014
500. 26867956
501. 26940579
502. 26968474
503. 27061932
504. 27107610
505. 27117516
506. 27199383
507. 27367359
508. 27456299
509. 27588751
510. 27638554
511. 27641453
512. 27648582
513. 27733271
514. 27859978
515. 27885969
516. 27889197
517. 27999418
518. 28041582
519. 28165571
520. 28181014
521. 28181963
522. 28238580
523. 28242397
524. 28279544
525. 28361527
526. 28390035
527. 28525644
528. 28719480
529. 28830891
530. 28891226
531. 28893705
532. 29066217
533. 29105820
534. 29193156
535. 29425537
536. 29512407
537. 29546772
538. 29665727
539. 29689743
540. 29855215
541. 29914835
542. 29958178
543. 30005979
544. 30168024
545. 30262170
546. 30293728
547. 30335833
548. 30481595
549. 30500642
550. 30525252
551. 30528961
552. 30570076
553. 30604166
554. 30627743
555. 30685808
556. 30739421
557. 30880803
558. 31215692
559. 31244374
560. 31300597
561. 31352911
562. 31379000
563. 31387422
564. 31387735
565. 31486593
566. 31489630
567. 31492632
568. 31541507
569. 31634606
570. 31634972
571. 31701380
572. 31735091
573. 31761874
574. 31800490
575. 31815899
576. 31818338
577. 31900609
578. 31978169
579. 32120280
580. 32152185
581. 32157910
582. 32253905
583. 32283170
584. 32339616
585. 32468147
586. 32476588
587. 32587073
588. 32597354
589. 32642096
590. 32676177
591. 32703168
592. 32734578
593. 32744334
594. 32925404
595. 32960848
596. 32985345
597. 32997191
598. 33030649
599. 33127247
600. 33155092
601. 33169648
602. 33306540
603. 33367573
604. 33694086
605. 33710161
606. 33718686
607. 33722419
608. 33777430
609. 33905849
610. 33917703
611. 33950754
612. 34001793
613. 34175956
614. 34293064
615. 34511570
616. 34643276
617. 34684057
618. 34712613
619. 34978721
620. 34991865
621. 35001802
622. 35169606
623. 35475427
624. 35476972
625. 35527180
626. 35758539
627. 35912672
628. 35959134
629. 35978182
630. 36004313
631. 36017820
632. 36210642
633. 36328817
634. 36569714
635. 36572322
636. 36639031
637. 36847159
638. 36861176
639. 37016194
640. 37231807
641. 37254629
642. 37357344
643. 37363978
644. 37509574
645. 37591312
646. 37593341
647. 37608678
648. 37655375
649. 37705250
650. 37805693
651. 37941512
652. 37965315
653. 38060973
654. 38166462
655. 38256349
656. 38490927
657. 38494399
658. 38581861
659. 38605511
660. 38622044
661. 38640999
662. 38720605
663. 38833346
664. 39064522
665. 39106314
666. 39289143
667. 39414915
668. 39428641
669. 39527930
670. 39555372
671. 39657908
672. 39658589
673. 39803105
674. 40214390

***PubMed* screening**

Criteria 1 = 593
Criteria 2 = 0
Criteria 3 = 71
Criteria 4 = 2
Inserted = 8
Additional relevant paper = 1 → PMID 17618813

***Cochrane Library* string**

ID	Search	Hits
#1	Neoplasms	110554
#2	cancer	243250
#3	tumor	89140
#4	tumour	89140
#5	Venous	36175
#6	Arterial	64384

**#7**	Vascular	67320
**#8**	Scaffold	1078
**#9**	Graft	33503
**#10**	Prosthesis	16617
**#11**	Arteriovenous Fistula	1607
**#12**	AVF	754
**#13**	Bridge Fistula	39
**#14**	Venous Access Device	844
**#15**	Central Venous Catheter	5132
**#16**	Peripherally Inserted Central Catheter	560
**#17**	PICC	596
**#18**	#1 OR #2 OR #3 OR #4	283102
**#19**	#5 OR #6 OR #7	141708
**#20**	#8 OR #9 OR #10	49598
**#21**	#19 AND #20	7734
**#22**	#11 OR #12 OR #13	1830
**#23**	#16 OR #17	778
**#24**	#22 OR #14 OR #15 OR #23	7453
**#25**	#18 AND #21 AND #24	44

***Cochrane Library* access date**
14th of January 2025

***Cochrane Library* results (44)**

1. CD001458
2. CD004588.PUB3
3. CD006406.PUB2
4. CD007921.PUB2
5. CD004082.PUB5
6. CD006142.PUB3
7. CD005341.PUB3
8. CD003298.PUB3
9. CD005540.PUB3
10. CD006247.PUB3
11. CD011319.PUB2
12. CD007888.PUB3
13. CD009904.PUB2
14. CD012779.PUB2
15. CD010649.PUB2
16. CD012368.PUB2
17. CD012726.PUB2
18. CD013641.PUB2
19. CD012920.PUB2
20. CD013486.PUB2
21. CD009898.PUB4
22. CD013751.PUB2
23. CD009759.PUB4
24. CD010185.PUB4
25. CD013201.PUB3
26. CD007315.PUB3
27. CD013074.PUB2
28. CD001888.PUB5
29. CD009535.PUB3
30. CD006536.PUB5
31. CN-00264605
32. CN-00328658
33. CN-00967365
34. CN-01011295
35. CN-01439733
36. CN-01876831
37. CN-01819628
38. CN-02293885
39. CN-02307783
40. CN-02405394
41. CN-02417390
42. CN-02527770
43. CN-02767514
44. D.O.I. → 10.1002/cca.1866

***Cochrane Library* screening**
Criteria 1 = 44
Criteria 2 = 0
Criteria 3 = 0
Criteria 4 = 0
Inserted = 0
